# Strigolactones optimise plant water usage by modulating vessel formation

**DOI:** 10.1038/s41467-025-59072-y

**Published:** 2025-04-28

**Authors:** Jiao Zhao, Dongbo Shi, Kiara Kaeufer, Changzheng Song, Dominik Both, Anna Lea Thier, Hui Cao, Linus Lassen, Xiaocai Xu, Yuki Hamamura, Laura Luzzietti, Tom Bennett, Kerstin Kaufmann, Thomas Greb

**Affiliations:** 1 Developmental Physiology, Centre for Organismal Studies, Heidelberg University, Heidelberg, Germany; 2 Genetics, Institute of Biochemistry and Biology, University of Potsdam, Potsdam, Germany; 3 RIKEN Center for Sustainable Resource Science, Yokohama, Japan; 4 Plant Cell and Molecular Biology, Institute of Biology, Humboldt-Universität zu Berlin, Berlin, Germany; 5 Max Planck Institute of Molecular Plant Physiology, Potsdam, Germany; 6 School of Biology, Faculty of Biological Sciences, University of Leeds, Leeds, UK

**Keywords:** Plant stem cell, Strigolactone, Drought

## Abstract

Wood formation is crucial for plant growth, enabling water and nutrient transport through vessel elements, derived from cambium stem cells (CSCs). CSCs produce vascular cell types in a bidirectional manner, but their regulation and cell fate trajectories remain unclear. Here, using single-cell transcriptome analysis in *Arabidopsis thaliana*, we reveal that the strigolactone (SL) signalling pathway negatively regulates vessel element formation, impacting plant water usage. While SL signalling is generally active in differentiating vascular tissues, it is low in developing vessels and CSCs, where it modulates cell fate decisions and drought response. SL-dependent changes in vessel element formation directly affect transpiration rates via stomata, underscoring the importance of vascular tissue composition in water balance. Our findings demonstrate the role of structural alignment in water-transport tissues under unstable water conditions, offering insights for enhancing drought resistance in plants through long-term modulation of vascular development.

## Abstract (web summary)

Vessel elements arise from cambium stem cells and support plant growth by supplying water and nutrients. This study reveals that strigolactone signalling inhibits vessel formation in response to drought, affecting transpiration and drought resistance.

## Introduction

Continuous growth and tissue formation are characteristic of plant development and important for aligning distinct body structures with changing environmental conditions. Cambium-driven radial growth of shoots and roots of dicotyledonous species is an important feature of this growth mode, and key for biomass production and for the long-term sequestration of CO_2_^1^. CSCs proliferate, usually providing wood (i.e. xylem) cells inwards and bast (i.e. phloem) cells outwards. Within xylem and phloem tissues, various cell types fulfil highly specialised functions like water transport by xylem vessel elements or sugar transport by phloem sieve elements^2^. However, mechanisms regulating CSC-associated cell fate decisions to cope with stressful environmental conditions are largely unknown.

Driven by transpiration at leaves and the resulting negative pressure in vessel elements, xylem-associated water transport is crucial for delivering water and dissolved minerals from the roots to the leaves, where photosynthesis occurs. Thus, disruptions in water transport, such as drought or embolism, can severely impair plant performance, reducing photosynthetic efficiency and metabolic rates, ultimately boosting drought-induced mortality^3^. The efficiency of water transport is a central parameter to ensure constant water supply under fluctuating water availability and, to a large extent, depends on the morphology and size of vessel elements. In fact, functions describing transpiration-based water loss indicate that the xylem is limiting for water transport when water is readily available^4^. Therefore, pathways actively modulating vessel element formation in response to alternating water availability are expected but have not been identified in the context of cambium-driven xylem formation.

Here, we provide cell-resolved transcriptomes of CSCs and all CSC-derived cell types generated by single-nucleus RNA-sequencing (snRNA-seq). Based on our droplet- and plate-based high-resolution transcriptome analysis, we identify SL signalling as a modulator of vessel formation and, thereby, as a module mediating structural adaptations to fluctuating water availability and drought stress.

## Results

### snRNA-seq analysis of radial growth

**Fig. 1:**
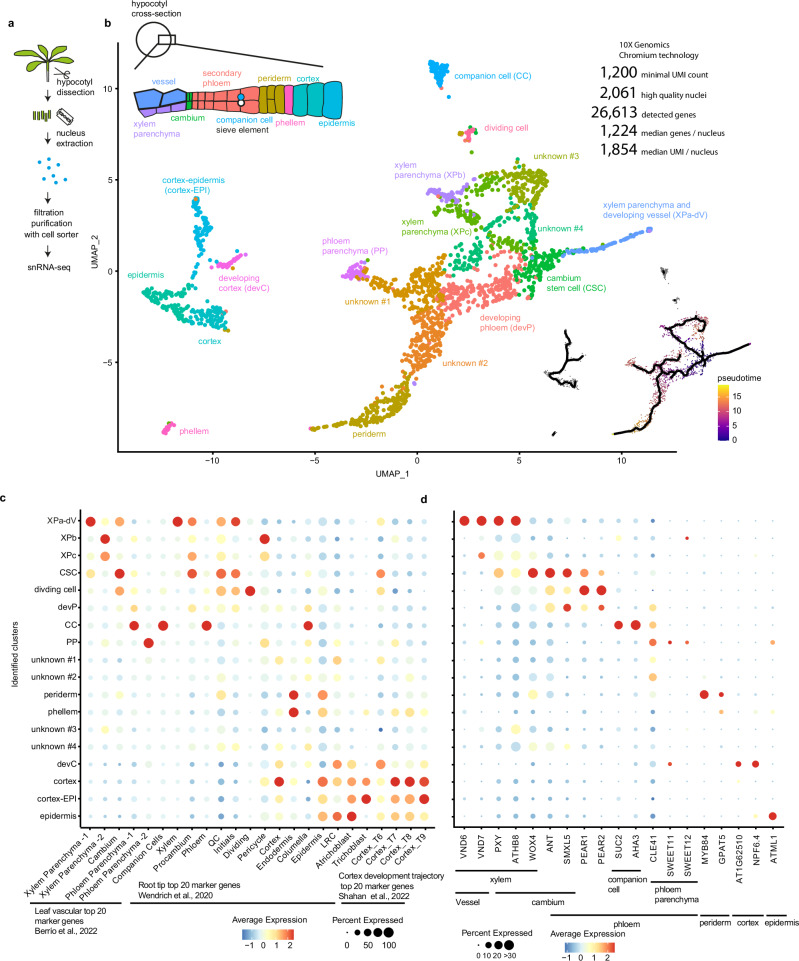
Identification of Arabidopsis hypocotyl cell types using 10x Chromium snRNA-seq. **a** Sample collection pipeline. **b** UMAP plot of 10x Chromium snRNA-seq analysis using 2,061 Arabidopsis hypocotyl nuclei organised in 18 clusters obtained through unsupervised clustering analysis. Differentiation trajectory based on pseudo-time analysis originating from CSCs is shown by black lines in the right-bottom. A scheme of major hypocotyl cell types is shown in the top left corner. **c**, **d** Dot plot showing the expression of tissue-specific and stage-specific marker genes identified in previous scRNA-seq analyses^8,9,71^ (**c**) and previously characterised tissue-specific marker genes (**d**) in the identified clusters (y-axis, shared between **c** and **d**). The size of the circles represents the percentage of cells with expression (percent expressed), whereas the colour indicates the scaled average expression (average expression). XPa-dV xylem parenchyma and developing vessels, XP xylem parenchyma, CSC cambium stem cells, devP developing phloem, CC companion cells, PP phloem parenchyma, devC developing cortex, cortex-EPI cortex-epidermis. Source data are provided as a Source Data file.

To comprehensively reveal cell states during radial plant growth, we established an snRNA-seq-based atlas of the Arabidopsis hypocotyl, the organ connecting shoot and root systems and being a hotspot for cambium-based organ growth^5^. To this end, nuclei were extracted, filtered, and purified via a cell sorter and processed by droplet-based single nucleus transcriptome analysis^6^ (Fig. 1a, Supplementary Fig. 1a). Demonstrating the success of our approach, at least 400 transcribed genes were detected in 4722 out of 6780 processed nuclei. From those, we kept 2061 high-quality nuclei in which at least 1200 unique molecular identifier (UMI) counts were identified for further analysis. Within these high-quality nuclei, we detected 26,613 genes as being transcribed, covering nearly 70% of the annotated genes for Arabidopsis^7^. On the median level, our analysis detected 1224 transcribed genes and 1854 UMIs for each nucleus (Supplementary Data 1).

By unsupervised clustering of the high-quality nuclei, we obtained 18 clusters, as visualised in a ‘uniform manifold approximation and projection’ (UMAP, Fig. 1b, Supplementary Data 2). Based on publicly available cell type-specific marker genes^8,9^, we annotated cluster identities representing all major cell types reported for the hypocotyl (Fig. 1c, d, Supplementary Data 3). These cell types included the cambium (see below) and the periderm, which is a secondary protective tissue characterised by *MYB84*, *GLYCEROL-3-PHOSPHATE SN-2-ACYLTRANSFERASE5* (*GPAT5*), and *WUSCHEL RELATED HOMEOBOX4* (*WOX4*) expression^10^. As an exception, phloem sieve elements were not detected, presumably due to the absence of nuclei in those cells. Annotations were verified by promoter reporter analyses of selected genes marking vascular clusters (Supplementary Fig. 2). Importantly, we identified a CSC-associated cluster containing 139 nuclei specifically expressing 151 genes including the known CSC markers *WOX4*^11–13^, *PHLOEM INTERCALATED WITH XYLEM* (*PXY*)^11,14,15^, *SUPPRESSOR OF MAX2 1-LIKE5* (*SMXL5*)^11,16^, *AINTEGUMENTA* (*ANT*)^17,18^, and *ARABIDOPSIS THALIANA HOMEOBOX8* (*ATHB8*)^19^ (p-value adjusted based on Bonferroni correction (p_adj) <0.01, Fig. 1d, Supplementary Data 2)^20^. Pseudotime analyses reconstructing developmental trajectories of vascular clusters revealed that vascular-related trajectories were linked to the CSC cluster (Fig. 1b). This was in remarkable contrast to previous analyses of procambium cells, the precursors of cambium cells during apical growth, which usually localise at the end of developmental trajectories^9^. Our trajectory analyses moreover allowed a more detailed clusterization along the differentiation path, resulting in several clusters specifically representing small sections of the path (Supplementary Fig. 3, Supplementary Data 2). Collectively, our results revealed that CSCs hold a specific transcriptome and reflected the central role of CSCs as the developmental origin of cells driving radial plant growth.

To increase the analytic depth of our CSC characterisation, we next applied the plate-based ‘vast transcriptome analysis of single cells by dA-tailing’ (VASA-seq) method^21^ capturing both polyadenylated RNA and non-polyadenylated RNA, and used fluorescence intensity of the cambium domain reporters *PXY*_*pro*_*:H4-GFP* and *SMXL5*_*pro*_*:H2B-RFP*^11^ during fluorescence-activated sorting to isolate 1134 cambium nuclei (Supplementary Fig. 1b, c). In addition, we isolated the same number of nuclei from the whole hypocotyl without *PXY*_*pro*_*:H4-GFP/SMXL5*_*pro*_*:H2B-RFP*-based sorting. In total, we processed 2,268 nuclei, resulting in 1559 high-quality nuclei showing at least 1,200 unique fragment identifier (UFI) counts/nucleus (Supplementary Fig. 4a, Supplementary Data 1). Overall, we detected transcripts of 33,195 genes, including the two reporter transgenes, covering more than 85% of the annotated genes in Arabidopsis. On the median level, our VASA-seq approach detected 2688 genes/nucleus and 5210 UFI/nucleus, which matched previous high-quality datasets for Arabidopsis nuclei^22^.

Unsupervised clustering of the VASA-seq processed nuclei resulted in 19 clusters, which we associated with distinct cell states using marker genes identified previously and during our droplet-based approach (Supplementary Fig. 4b, c). Again, most hypocotyl cell types were represented by a cluster in the VASA-seq dataset with a lower relative size of ‘periderm’ or ‘developing vessel’ clusters than obtained by our droplet-based approach, presumably due to the depletion of those nuclei during the cambium-centred nucleus selection (Supplementary Data 2). Moreover, CSC marker genes were expressed this time in two clusters representing CSCs (‘VASA_CSC’ cluster, 94 nuclei) and dividing CSCs (‘VASA_dividing’ cluster, 66 nuclei) (Supplementary Fig. 4c) based on transcript abundance of genes previously associated with dividing root cells^9^. Supporting the conclusion that these were CSC nuclei, both clusters showed high GFP (*PXY*_*pro*_*:H4-GFP*) and RFP (*SMXL5*_*pro*_*:H2B-RFP*) fluorescence intensities^11^ (Supplementary Fig. 5a). We furthermore identified 226 and 405 marker genes for each CSC cluster, respectively (p_adj <0.01, Supplementary Data 2) demonstrating that the VASA-seq analysis substantially increased analytical depth. Moreover, 119 of 151 (72%) CSC marker genes identified by our droplet-based approach were included in the groups of CSC and/or dividing CSC marker genes (Supplementary Fig. 5b) and could be confirmed by expression analyses in plants (Supplementary Fig. 6), suggesting that results from both approaches were robust.

### SL signalling activity within radial growth

**Fig. 2:**
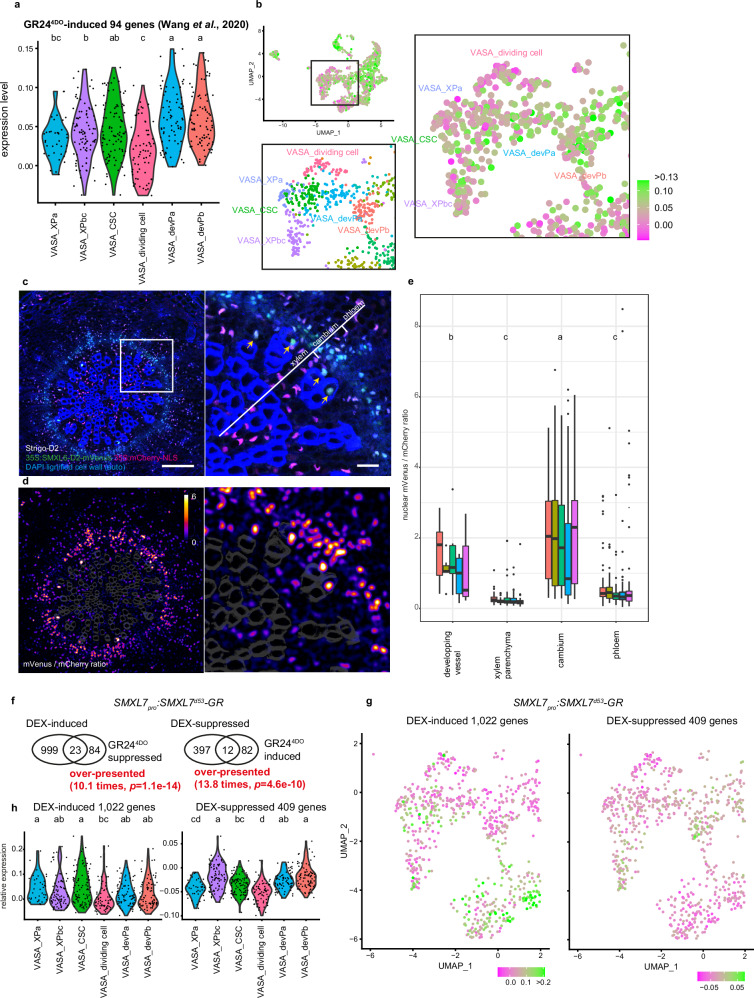
Strigolactone (SL) signalling in the radially expanding hypocotyl. **a**, **b** Violin plot (**a**) and UMAP visualisation (**b**) of expression profiles of 94 GR24^4DO^-induced genes^25^ in cambium-related cell clusters identified by VASA-seq. Statistical groups determined by the Steel-Dwass test for multiple comparisons are indicated by letters (*p* < 0.05). Gene lists used in this analysis can be found in Supplementary Data 3. A close-up of the UMAP originated from the VASA-seq cluster identification (Supplementary Fig. 4) is shown as a reference. **c** Maximum intensity projection of confocal microscopy images of 5-week-old hypocotyl cross-sections expressing Strigo-D2 (*35S*_*pro*_*:SMXL6-D2-mVenus_35S*_*pro*_*:mCherry-NLS*) as an indicator of SL signalling. mVenus and mCherry signals are shown in green and magenta, respectively. Hoechst33342 was used to stain nuclei, shown in blue, together with the autofluorescence of lignified cell walls in vessel elements. Yellow arrowheads indicate the nuclei of developing vessel elements. Size bars represent 100 µm and 20 µm on the left and right, respectively. **d** Ratiometric images of mVenus / mCherry signals after image processing (see “Methods” section). The colour bar indicates the scale of ratio values from 0 to 6. A higher ratio indicates lower SL signalling levels. **e** Box plot showing nuclear fluorescence intensity ratio of mVenus and mCherry in different vascular tissues. Data from one individual plant are shown in the same colour (*n* = 5). The total number of nuclei analysed for each tissue type of the five plants was 42 (developing vessels), 389 (xylem parenchyma), 903 (cambium), and 411 (phloem). Statistical groups are indicated by letters and assessed by a one-way ANOVA with post-hoc Tukey-HSD (95% CI) on the mean ratio value of each tissue type in each individual plant. **f** Venn diagram showing the overlap of *SMXL7*_*pro*_*:SMXL7*^*d53*^*-GR* DEX-induced genes and GR24^4DO^-suppressed genes, and of *SMXL7*_*pro*_*:SMXL7*^*d53*^*-GR* DEX-suppressed genes and GR24^4DO^-induced genes. Results of Fisher’s exact test are shown. **g**, **h** UMAP visualisation (**g**) and violin plot (**h**) of expression profiles of DEX-induced and DEX-suppressed genes in the cambium-related cell clusters identified by VASA-seq. Statistical groups determined by the Steel-Dwass test for multiple comparisons are indicated by letters (*p* < 0.05). Source data are provided as a Source Data file. For box plot definition, please see “Statistics and reproducibility” section.

Demonstrating their relevance, our cell-resolved transcriptome data recapitulated xylem-associated auxin signalling and phloem-associated cytokinin signalling^23^ when we monitored transcript abundance of respective hormone-responsive genes^24^ within identified clusters (Supplementary Fig. 7, Supplementary Data 3). Interestingly, when we mapped the activity of 94 genes induced by the synthetic SL analogue GR24^4DO^^25^, we found that the expression of these genes was particularly low in the cluster representing dividing CSCs and in a subset of the xylem cell clusters, presumably representing differentiating vessel elements (‘VASA_XPa’, Fig. 2a, b). Indeed, when analysing SL signalling levels by the genetically encoded SL signalling sensor Strigo-D2^26^, we found that SL-signalling activity was lower in CSCs and in developing vessel elements compared to phloem and xylem parenchyma cells (Fig. 2c–e). To find further support for this conclusions and to map the activity of SL-dependent genes in radially growing organs, we expressed an SL-resistant version of the proteolytic target of the SL-signalling pathway SMXL7 (SMXL7^d53^)^27^ fused to the dexamethasone (DEX)-inducible GLUCOCORTICOID RECEPTOR (GR) under the control of the endogenous *SMXL7* promoter (*SMXL7*_*pro*_*:SMXL7*^*d53*^*-GR*). When performing transcriptional profiling of hypocotyls five hours after DEX application, we identified 1,022 DEX-induced and 409 DEX-suppressed genes (Supplementary Data 4) significantly overlapping with previously-reported GR24^4DO^-suppressed and GR24^4DO^-induced genes^25^, respectively (Fig. 2f). This suggested that our analysis successfully identified genes downstream of SL signalling in the Arabidopsis hypocotyl. In line with results obtained using the Strigo-D2 sensor, in both VASA and 10x snRNA-seq datasets, DEX-suppressed genes showed significantly lower expression in clusters representing developing vessels and cambium stem cells when compared to xylem parenchyma and phloem clusters (Fig. 2g, h, Supplementary Fig. 8). Taken together, these results indicated that SL signalling levels are relatively low in developing vessels and in CSCs.

SLs are a class of carotenoid-derived phytohormones, modulating various processes including shoot branching and drought resistance^28,29^. SL molecules are perceived by the α/β hydrolase DWARF14 (D14), inducing interaction with the F-box protein MORE AXILLARY GROWTH2 (MAX2) and thereby promoting degradation of the transcriptional regulators SMXL6, SMXL7, and SMXL8^25,30,31^. Using fluorescent promoter reporters, we found that the genes encoding these signalling components were widely active in the hypocotyl with a particular focus on the xylem (Supplementary Fig. 9): The activity of the *MAX2* reporter was detected in developing xylem vessels, xylem parenchyma cells, cambium cells and phloem parenchyma cells, whereas *D14* reporter activity was only detected in xylem parenchyma cells and phloem parenchyma cells. *SMXL6*, *SMXL7*, and *SMXL8* reporter activities were all detected in xylem parenchyma cells, with *SMXL7* also being detected in developing vessels (Supplementary Fig. 9). The transcripts of several SL biosynthetic genes were also widely detected both in xylem parenchyma cells and developing vessels (Supplementary Fig. 10). Our analyses thus associated SL signalling components with differentiating and differentiated vascular tissues and were in line with a regulatory role of the pathway in their formation.

### SL-signalling suppresses vessel formation

**Fig. 3:**
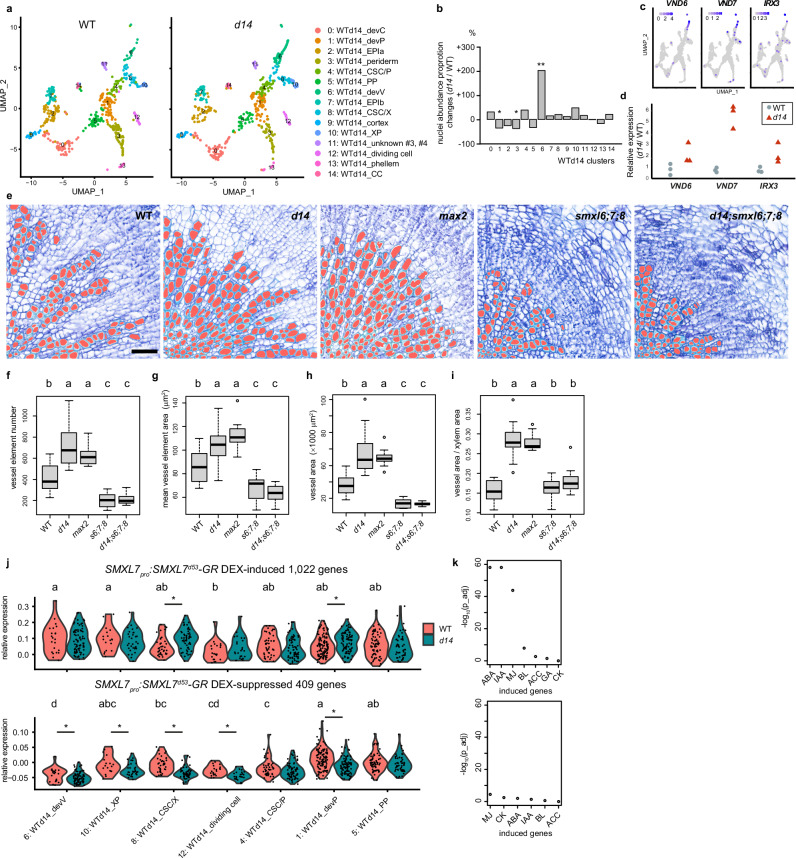
Effect of SL signalling on vessel development. **a** UMAP plot of 10x Chromium snRNA-seq analysis using 500 hypocotyl nuclei organised in 15 clusters obtained through unsupervised clustering. 500 nuclei were randomly resampled from 684 (WT) and 684 (*d14*) nuclei, respectively. Abbreviations for annotated clusters are devV developing vessel, XP xylem parenchyma, CSC/X cambium stem cell/xylem, CSC/P cambium stem cell/phloem, devP developing phloem, CC companion cell, PP phloem parenchyma, devC developing cortex, EPI epidermis. **b** Changes in relative nuclei abundance for each cluster in *d14* mutants compared to wild type. Fisher’s exact test was used to determine statistical differences in each cluster between genotypes. *p < 0.05, **p < 0.01. *p* = 1.0e-03 (cluster 1:WTd14_devP), *p* = 3.1e-03 (cluster 3:WTd14_periderm), *p* = 2.9e-07 (cluster 6:WTd14_devV). **c** Expression of the developing vessel marker genes *VND6*, *VND7* and *IRX3* in the UMAP plot shown in a. **d** Relative transcript abundance of *VND6*, *VND7* and *IRX3* in *d14* mutants compared to wild type, normalised to the *EF-1a* reference gene in qRT-PCR experiments. *n* = 3 biological replicates. **e** Toluidine-blue stained hypocotyl cross-sections from five week-old wild type, *d14*, *max2*, *smxl6;7;8* and *d14;smxl6;7;8* plants. Vessels were automatically identified using ImageJ and subsequent manual correction (marked in red). Scale bar represents 50 μm. **f**–**i** Quantification of the vessel element numbers per section (**f**), the average area of individual vessel elements (**g**), the total vessel area per section (**h**) and the ratio between the vessel element area and the total xylem area (**i**) in different genotypes. *n* = 15 (WT), 15 (*d14*), 13 (*max2*), 10 (*smxl6;7;8*), 14 (*d14;smxl6;7;8*) plants for each genotype. Statistical groups are indicated by letters and determined by a one-way ANOVA with post-hoc Tukey-HSD (95% CI). **j** Violin plot of expression profiles of DEX-induced and DEX-suppressed genes (SMXL7^d53^-GR) in the cambium-related cell clusters identified by the WT-d14 10x snRNA-seq dataset. Statistical groups for wild-type expression patterns determined by the Steel-Dwass test for multiple comparisons are indicated by letters (*p* < 0.05). Asterisks indicate significant difference of transcript abundance between wild type and *d14* mutants in each cluster, respectively (Steel-Dwass test, *p* < 0.05). *p* = 1.6e-03 (cluster 8, induced), 2.2e-02 (cluster 1, induced), 4.1e-02 (cluster 6, suppressed), 3.1e-02 (cluster 10, suppressed), 4.3e-07 (cluster 8, suppressed), 2.4e-03 (cluster 12, suppressed), 5.4e-07 (cluster 1, suppressed). *p*-values are also shown in the Source Data file. **k** Enrichment of DEX-induced (top) or DEX-suppressed (bottom) genes in phytohormone responsive gene lists curated from Nemhauser et al., 2006^24^. Bonferroni corrected *p-*value of Fisher’s one-tailed test is shown in a negative log_10_ format. IAA indole 3 acetic acid (auxin), CK zeatin (cytokinin), ABA abscisic acid, ACC 1-amino-cyclopropane-1-carboxylic acid (ethylene precursor), BL brassinosteroid, MJ methyl jasmonate (jasmonate), GA gibberellic acid (gibberellin). Source data are provided as a Source Data file. For box plot definition, please see “Statistics and reproducibility” section.

To reveal a possible function of the SL-signalling pathway in vascular tissue formation, we next compared hypocotyls from *d14* mutants and wild type by again applying droplet-based snRNA-seq. After clustering and cluster annotation based on our initially identified genes marking the different hypocotyl cell types (Supplementary Fig. 11, Supplementary Data 1), we found that cell composition and transcriptional states were highly similar between the two genotypes (Fig. 3a). Interestingly, as the most prominent difference, we detected a three-fold increase in the abundance of nuclei from developing vessel elements (WTd14_devV) in *d14* mutants (Fig. 3b, Supplementary Fig. 11). Those were identified based on the expression of the *VASCULAR-RELATED NAC-DOMAIN6* (*VND6*), *VND7*^32,33^ and *IRREGULAR XYLEM3* (*IRX3)*^34^ and other xylem marker genes (Fig. 3c, Supplementary Fig. 11d–i). In line with an increased number of vessel elements, transcripts of *VND6*, *VND7* and *IRX3* were more abundant in *d14* mutants (Fig. 3d). Indeed, histological analyses revealed a higher density and increased size of vessel elements in both the SL signalling mutants *d14* and *max2* (Fig. 3e–i) and the SL biosynthesis mutant *max1* (Supplementary Fig. 12a–c). This alteration was not observed in *BRANCHED1* (*BRC1*)-defective plants (Supplementary Fig. 12d–f), which, similar to *d14*, *max1*, and *max2* mutants, show increased branching^35^, arguing against the possibility that increased shoot formation causes enhanced vessel formation as a secondary effect. Moreover, *KAI2*-deficient plants impaired in KARRIKIN signalling, which, like SL signalling, involves the F-box protein MAX2^36^, did not show enhanced vessel formation (Supplementary Fig. 12g–i), demonstrating that altered vessel formation specifically depends on altered SL signalling.

**Fig. 4:**
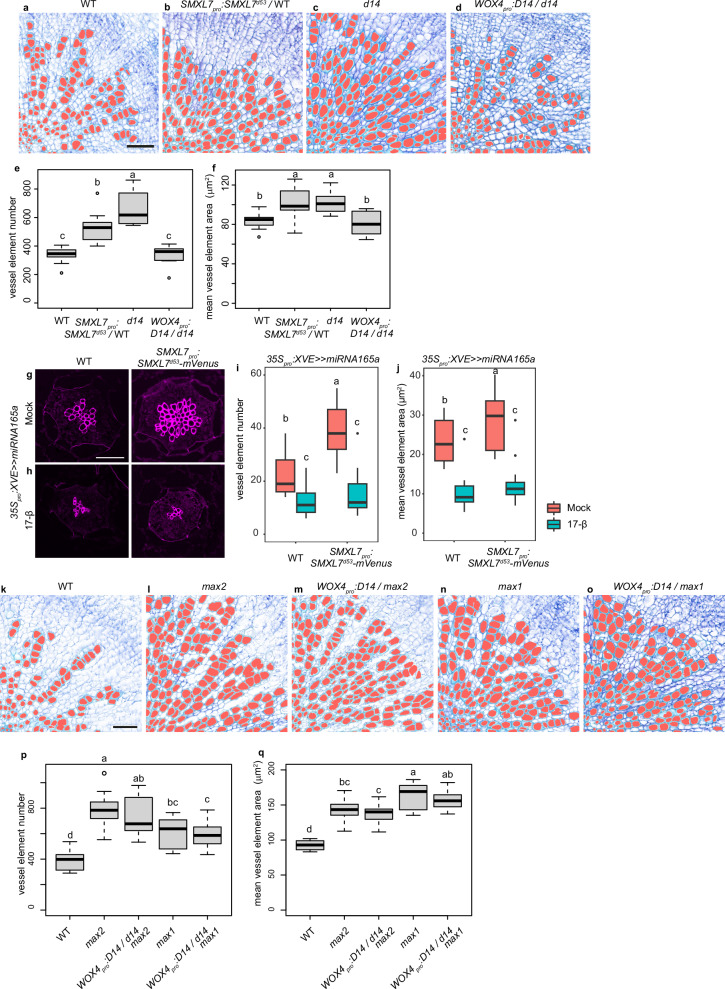
Analysis of *SMXL7*_*pro*_*:SMXL7*^*d53*^*-mVenus*, *WOX4*_*pro*_*:D14/d14*, *WOX4*_*pro*_*:D14/max2*, and *WOX4*_*pro*_*:D14/max1* plants. **a**–**d** Toluidine blue-stained hypocotyl cross-sections from 5-week-old wild type (**a**, **b**) and *d14* (**c**, **d**) plants carrying a *SMXL7*_*pro*_*:SMXL7*^*d53*^*-mVenus* (**b**) or *WOX4*_*pro*_*:D14* (**d**) transgene. **e**, **f** Quantification of vessel element number per section (**e**) and mean vessel element area size per section (**f**) comparing wild type, *d14*, lines carrying *WOX4*_*pro*_*:D14* transgenes in the *d14* background and lines carrying a *SMXL7*_*pro*_*:SMXL7*^*d53*^*-mVenus* transgene. *n* = 11 (WT), 13 (*SMXL7*_*pro*_*:SMXL7*^*d53*^*-mVenus*/WT), 12 (*d14*) and 9 (*WOX4*_*pro*_*:D14*/d14) plants for each genotype. This assay was conducted in parallel with the *brc1* vessel element analysis, and both share the same wild-type data. **g**, **h** Basic Fuchsin-stained hypocotyl cross-sections from 2 week-old *35S*_*pro*_*:XVE* > *>miRNA165a* transgenic plants in wild type (**g**) and *SMXL7*_*pro*_*:SMXL7*^*d53*^*-mVenus* background (**h**), following *miRNA165a* induction by 17β-Estradiol (17-β) in 5-day-old seedlings. **i**, **j** Quantification of vessel element number per section (**i**) and mean vessel element area size per section (**j**). *n* = 17 (WT, Mock), 22 (WT, 17-β), 17 (*SMXL7*_*pro*_*:SMXL7*^*d53*^*-mVenus*, Mock) and 25 (*SMXL7*_*pro*_*:SMXL7*^*d53*^*-mVenus*, Mock, 17-β) plants for each group. **k**–**o** Toluidine blue-stained hypocotyl cross-sections from five-week-old wild type (**k**), *max2* (**l**), *max2* plants carrying a *WOX4*_*pro*_*:D14* transgene (**m**), *max1* (**n**) and *max1* plants carrying a *WOX4*_*pro*_*:D14* transgene (**o**). **p**, **q** Quantification of vessel element number per section (**p**) and mean vessel element area size per section (**q**). *n* = 8 (WT), 11 (*max2*), 11 (*WOX4*_*pro*_*:D14/max2*), 10 (*max1*) and 13 (*WOX4*_*pro*_*:D14/max1*) plants for each genotype. Statistical groups are indicated by letters and were determined by a one-way ANOVA with post-hoc Tukey-HSD (95% CI). Vessel elements were automatically identified using ImageJ and subsequent manual correction (marked in red) (a–d, k–o). Scale bar represents 50 μm. Source data are provided as a Source Data file. For box plot definition, please see “Statistics and reproducibility” section.

In contrast to the SL signalling and biosynthesis mutants, the number and size of vessel elements was decreased in *smxl6;smxl7;smxl8* (*smxl6;7;8*), *d14*;*smxl6;7;8* (Fig. 3e–i) and *max2;smxl6;7;8* mutants (Supplementary Fig. 12j, k) demonstrating that increased vessel formation in SL-related mutants depends on the proteolytic targets of the SL signalling pathway. To this effect, mostly *SMXL7* and *SMXL8* contributed in a redundant fashion, as no other *max2;smxl* double or triple mutant combination than *max2;smxl7;8* showed a mild reduction in vessel density in comparison to *max2* (Supplementary Fig. 12j, k). Further supporting a positive role of the proteolytic targets of SL-signalling in vessel formation, expressing SMXL7^d53^, the SL-resistant version of SMXL7^27^, under the control of the *SMXL7* promoter (*SMXL7*_*pro*_*:SMXL7*^*d53*^*-mVenus*) was sufficient to promote cambium-associated vessel formation (Fig. 4a, b, e, f). Moreover, SMXL7^d53^-GR-inducible genes were significantly upregulated and SMXL7^d53^-GR-repressible genes were significantly downregulated in *d14* mutants in several vascular-related clusters (Fig. 3j), indicating that SL signalling indeed regulates gene expression in vascular cells. In accordance with previous reports that SL signalling negatively regulates the expression of auxin-dependent genes^25^, we also found that auxin-inducible genes were significantly enriched in the group of SMXL7^d53^-GR-inducible genes together with abscisic acid (ABA) and methyl jasmonate (MJ)-inducible genes (Fig. 3k, Supplementary Data 3). This result suggested a systematic negative effect of SL signalling on auxin, ABA and jasmonate signalling. With three SMXL7^d53^-GR-inducible and 13 SMXL7^d53^-GR-repressible genes specifically expressed in xylem parenchyma (VASA_XPb cluster), including the auxin transporter genes *PIN-FORMED 3* and *LIKE AUXIN RESISTANT 2* (Supplementary Data 2, Supplementary Data 4), we also identified candidates regulating the ratio of vessels and xylem parenchyma downstream of SL signalling. Along these lines, when reducing the activity of the HD ZIP-III transcription factor family, an established group of auxin-dependent xylem regulators, by inducing *miRNA165a* expression targeting all five *HD ZIP-III* mRNAs^17,37^, the difference in vessel formation observed between wild type and the *SMXL7*_*pro*_*:SMXL7*^*d53*^*-mVenus* line, disappeared (Fig. 4 g–j). This argued for an essential role of the HD ZIP-III transcription factors downstream of the SL signalling pathway and for an influence of SL signalling on canonical regulators of xylem development.

Furthermore, indicating a vascular-associated role of SL signalling, *d14* mutants did not show an increased vessel phenotype when they carried a transgene expressing *D14* under the control of the cambium- and early xylem-specific *WOX4* promoter (*WOX4*_*pro*_*:D14*) (Fig. 4a, c–f). The same transgene had no effect in *max2* or *max1* mutants, demonstrating that the effect of *D14* depended on endogenous SL signalling and biosynthesis (Fig. 4k–q). Together with the observation that application of GR24^4DO^ specifically decreased vessel formation in wild type and *max1* but not in *d14* (Supplementary Fig. 13), we concluded that SL-signalling negatively regulates vessel formation during radial plant growth in cambium-derived cells and in an *SMXL6;7;8*-dependent fashion.

### Vessel development influences transpiration

Relative water content is reduced in *d14* and *max2* mutants^38–40^ and increased in *smxl6,7,8* triple mutants when grown in water-deprived pots^41^, suggesting that SL signalling optimises water usage. Confirming this role, water usage was increased in *d14* and *max2* mutants and reduced in *smxl6;7;8* mutants (Supplementary Fig. 14a). ABA-induced closure of stomata, the main transpiration site in plants, is slower in SL signalling deficient mutants and proposed as one cause for their reduced drought resistance^38–40^. Indeed, stomatal conductance was elevated in *d14* and *max2* mutants and lower in *smxl6;7;8* mutants compared to wild-type plants under both well-watered and water-deficiency conditions (Supplementary Fig. 14b), demonstrating that transpiration is reduced by SL signalling. In contrast, *brc1* mutants showed a stomatal conductance like wild type again demonstrating that enhanced branching does not cause higher transpiration (Supplementary Fig. 14c). Moreover, we found a similar reduction of stomatal conductance in *d14;smxl6;7;8* and *max2;smxl6;7;8* mutants compared to *smxl6;7;8* mutants (Supplementary Fig. 14c) indicating that D14 and MAX2 require their downstream targets SMXL6, SMXL7 and SMXL8 for affecting transpiration. Interestingly, we found that *d14* mutants carrying a *WOX4*_*pro*_*:D14* transgene showed normal transpiration rates and no altered stomata density (Supplementary Fig. 15a, b), indicating that this effect depended on SL signalling in vascular tissues. In accordance with previous reports^39,40^, stomata density was not changed in *d14* and *max2* mutants and only slightly enhanced in *smxl6;7;8* mutants (Supplementary Fig. 15b–e). Moreover, cuticle thickness was comparable between wild type and *d14* (Supplementary Fig. 15f), arguing against altered stomata density or cuticle defects as reasons for altered transpiration in SL-related mutants.

**Fig. 5:**
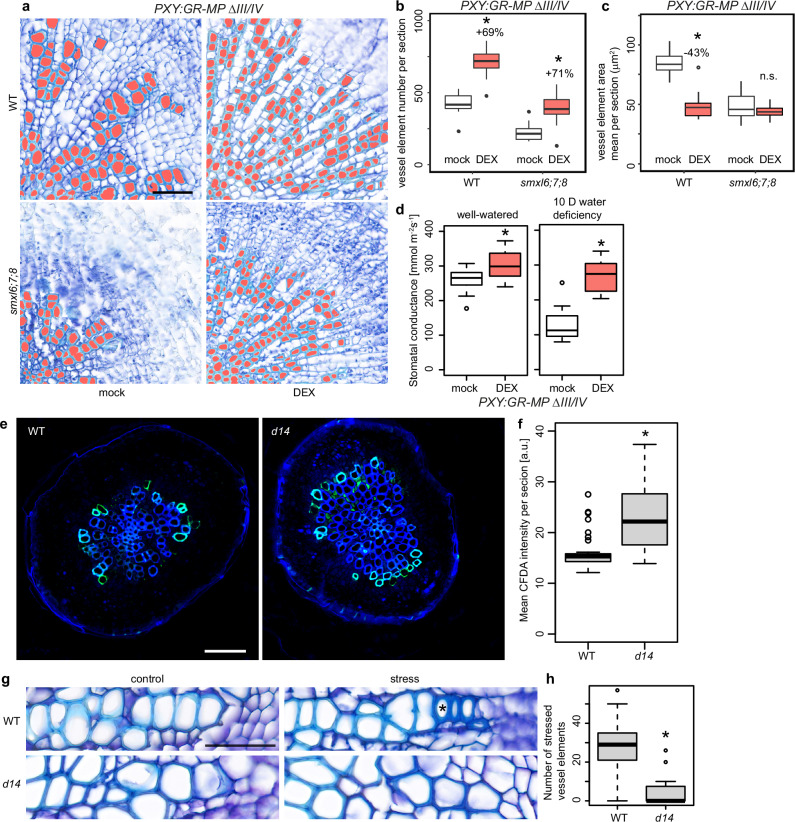
Effect of altered vessel formation on water usage and stomatal conductance. **a** Toluidine blue-stained hypocotyl cross-sections of five-week-old plants carrying a *PXY*_*pro*_*:GR-MPΔIII/IV* transgene in wild type or in *smxl6;7;8* triple mutants. Plants were treated with mock or DEX solutions for the last two weeks. Vessels were automatically identified using ImageJ and subsequent manual correction (marked in red). Scale bar represents 100 μm. **b**, **c** Vessel element numbers per section (**b**) and individual vessel element area mean per section (**c**) in response to DEX treatment comparing wild type and *smxl6;7;8* mutants as shown in (**a**). *n* = 12 (WT, Mock), 16 (WT, DEX), 14 (*smxl6;7;8*, Mock), 16 (*smxl6;7;8*, DEX) plants for each group. Asterisks indicate *p* < 0.01 in the post-hoc Bonferroni correction after one-way ANOVA for the effect of treatment for each genotype. *p* = 6.8e-09 (WT, **b**), *p* = 3.6e-05 (*smxl6;7;8*, **b**), *p* = 2.9e-09 (WT, **c**), *p* = 0.35 (*smxl6;7;8*, **c**). **d** Quantification and comparison of stomatal conductance in *PXY*_*pro*_*:GR-MPΔIII/IV* plants treated with mock or DEX solution under well-watered or water-deficiency (10 days) conditions. Stomatal conductance of three leaves was measured per plant using an SC1 Leave prometer. *n* = 15 plants for each condition. The asterisk indicates significance when the two-sided Welch’s t-test was applied, *p < 0.01, *p* = 2.8e-03 (well-watered), *p* = 4.9e-09 (10 days water deficiency). **e** Hypocotyl cross sections of wild type and *d14* plants after CFDA application. Xylem autofluorescence shown in blue and CFDA-derived signal in green. **f** Quantification of CFDA signal intensity between WT and *d14* in hypocotyl cross sections shown in (**e**). Scale bar indicates 50 µm. *n* = 33 plants (wild type) and 23 plants (*d14*). *p* = 6.5e-05 (two-sided Welch’s *t*-test). **g** Toluidine blue-stained hypocotyl vessel elements of five-week-old plants subjected to a soil-based water deficiency treatment. Vessels were automatically identified using VesselWizard and subsequent manual correction. Stress-related vessels are marked with asterisks. Scale bar represents 100 μm. **h** Quantification of stressed vessel element number between wild type and *d14*. The asterisk indicates significance when the two-sided Welch’s t-test was applied, *p < 0.01, *p* = 2.1e-06. *n* = 21 plants (wild type) and 15 plants (*d14*). Source data are provided as a Source Data file. For box plot definition, please see “Statistics and reproducibility” section.

Further supporting an impact of vessel development on water usage, induction of a DEX-inducible and auxin-insensitive version of the AUXIN RESPONSE FACTOR (ARF) transcription factor MONOPTEROS (MP, also known as ARF5) expressed under the control of the cambium-associated *PXY* promoter (*PXY*_*pro*_*:GR-MPΔIII/IV*^11,42,43^) resulted in a significant increase of vessel formation (Fig. 5a, b) and, at the same time, in elevated stomatal conductance in both well-watered and water deficiency condition (Fig. 5d). This effect was observed although stomata density was not significantly affected upon induction (Supplementary Fig. 15g, h). Interestingly, in wild type activation of auxin signalling by inducing MPΔIII/IV increased vessel number but reduced their size, while in *smxl6;7;8* mutants, only the vessel number increased (Fig. 5a–c). This observation indicated that, in contrast to a common but independent effect of SL and auxin signalling on vessel number, both pathways have an interconnected effect on vessel size. Based on these findings, we rationalised that SL signalling restricts water usage in plants by modulating vessel formation and, consequently, water transport capacity. Supporting this idea, application of carboxyfluorescein diacetate (CFDA) to roots, used to study long distance transport along the xylem^44^, resulted in higher fluorescence intensity in hypocotyls of *d14* mutants than in wild type (Fig. 5e, f).

To further explore the effect of reduced water availability on vessel formation and the role of SL-signalling in this process, we subjected wild-type and *d14* plants for two weeks to a soil-based water deficiency treatment. As a result, smaller vessels with a thicker cell wall were formed in wild type, while only very few of those were formed in *d14* mutants (Fig. 5g, h). Likewise, when we reduced water availability for plants by adding 9% polyethylene glycol (PEG_8000_) to hydroponic cultures^45^, fewer vessel elements with thickened cell walls were generated in *d14* than in wild type (Supplementary Fig. 14d, e). These observations showed that SL signalling promotes structural changes in vessel elements when plants experience water deficiency.

## Discussion

Taken together, by starting off with a cell-resolved transcriptome analysis of radially growing Arabidopsis organs, we revealed that SL signalling modifies the number and size of vessel elements produced during cambium-dependent radial growth. The possibility to modulate vessel anatomy in response to environmental cues like drought^46^, can be assumed to be an important fitness trait and has, so far, been described as being mediated by ABA signalling during longitudinal growth^47^. We did, however, neither observe any alteration of primary vessel element formation in primary *d14* roots (Supplementary Fig. 16a), nor an increase of cambium-derived vessel formation in *aba deficient 2* (*aba2*) mutants (Supplementary Fig. 16d, f), impaired in ABA biosynthesis^48^ and showing higher stomatal conductance^49^. Moreover, the formation of cambium-derived vessel elements was not altered in mutants displaying an increased stomatal density and a resulting increase in stomatal conductance^50,51^ (Supplementary Fig. 16e, f), arguing against a positive effect of enhanced transpiration on vessel formation. These findings suggest that ABA- and SL-signalling pathways fulfil distinct functions with regard to their effect on vessel formation during primary and secondary development, respectively. Based on the observation that increased vessel formation by enhancing auxin signalling also leads to enhanced transpiration, there is indeed the possibility that increased stomatal conductance in SL-related mutants is a result of enhanced vessel element formation and an associated increase in water transport along the vascular system.

Interestingly, SL biosynthesis is enhanced in rice roots in drought conditions^52^ indicating that the pathway has the potential to lead to structural changes when plants experience growth, to possibly anticipate harsh conditions in the future. Depending on distinct ecological or agricultural niches plants have adapted to, it may be advantageous to establish a weaker or stronger impact of SL signalling on vessel element formation to cope with distinct environmental regimes.

## Methods

### Plant material

All plant lines used in this study were *Arabidopsis thaliana* (L.) Heynh. plants of the accession Columbia (Col-0). The single and higher order mutants of *d14-1* (WiscDsLoxHs137_07E), *smxl6-4* (SALK_049115), *smxl7-3* (WiDsLox339_C04), *smxl8-1* (SALK_025338C), *max1-1*, and *max2-1* were obtained from Dave Nelson^53,54^ (UC Riverside, US) and Ottoline Leyser^55^ (SLCU, Cambridge, UK). The *35S*_*pro*_*:XVE* > *>miRNA165a* line was received from Ari Pekka Mähönen^17^ (University of Helsinki, Finland). The *brc1-2* (SALK_091920) mutant was ordered from NASC and was characterised previously^35,56–58^. *Strigo-D2*^26^, *PXY*_*pro*_*:ER-ECFP-HDEL*^59^, *PXY*_*pro*_*:H4-GFP;SMXL5*_*pro*_*:H2B-RFP*^11^, *SMXL7*_*pro*_*:SMXL7*^*d53*^*-VENUS*^27^, *PXY*_*pro*_*:Myc-GR-bdl*^42^, *MP*_*pro*_*:ER-EYFP-HDEL*^42^, and *PXY*_*pro*_*:GR-MPΔIII/IV*^42^ transgenic lines were described previously. Other transgenic lines were generated through the floral dipping method using *Agrobacterium tumefaciens*^60^. The *aba2-11* mutant^48^ was obtained from Mikael Brosché (University of Helsinki, Finland). *epf1-1* (SALK_137549)^61^ and *epf2-3* (SALK_047918)^62^ single and double mutants and *tmm-1* (SALK_011958)^63^ mutants were kindly donated by Christopher Grefen (University of Bochum, Germany). *PFA1*_*pro*_*:PFA1-GFP*^64^ lines were obtained from Tatsuo Kakimoto (Osaka University, Japan).

### Vector construction

The *WOX4*_*pro*_*:D14* (*pVJ13*) construct was generated using In-Fusion cloning (Takara Bio) using the amplified *D14* coding region and BamHI-digested *pTOM49*^13^. Other plasmids were generated using the GreenGate cloning system^65,66^. *SMXL7*^*d*53^ in pGGC (*pJZ60*) was mutagenised based on the template of the *SMXL7* CDS in pGGC (*pKR07*) according to the manual of the QuickChange Site-Directed Mutagenesis Kit (Agilent, Santa Clara, USA). See Supplementary Data 5 for oligo sequences used for molecular cloning and Supplementary Data 6 for each module used during the cloning process.

### Growth conditions

Arabidopsis seeds were surface sterilised by 70% ethanol containing 0.02% Tween-20, stratified at 4 °C for 2-3 days in the dark, and sown on 0.8% w/v agar in 1/2 Murashige and Skoog (MS) medium supplemented with 1% w/v sucrose. Seedlings were grown in short-day conditions (SD; 10 h light and 14 h darkness) at 21–22 °C. For morphological observations and reporter activity analyses, five-day-old seedlings were transferred to pots filled with a 4:1 mixture of soil and vermiculite. After 21 days, plants were transferred to long day conditions (LD; 16 h light and 8 h darkness) at 21–22 °C. Plants for stomatal conductance measurements were grown for six weeks in SD conditions (8.5 h light and 15.5 h darkness).

### Single-nucleus RNA-seq analysis

Hypocotyls were dissected and collected in petri dishes incubated on ice. 2 ml of 1× nuclei isolation buffer (CelLytic™ PN Isolation/Extraction Kit, Sigma #CELLYTPN1) supplemented with 20 µl RiboLock RNase inhibitor 40 U/µL (Thermo Fisher #EO0381) and Hoechst 33342 at 10 µg/ml final concentration were prepared and a minimum amount of buffer was applied to submerge the collected hypocotyls^10^. Hypocotyls were chopped using razor blades (Wilkinson Sword) for up to 5 min and then gently shaken at 4 °C for 15 min. Samples were then filtered through a 50 µm filter (CellTrics, Sysmex #04-004-2327) and passed to a low-protein-binding tube (Eppendorf #0030108132).

For 10x genomics application, 50,000 nuclei were sorted into 33 µl collection buffer (10 µl PBS (Corning, #21-040-CV), 5 µl BSA (Thermo Fisher, #AM2616), 6 µl RNAse inhibitor (Thermo Fisher, #AM2682), 12 µl RNAse inhibitor (Thermo Fisher, #AM2694)), modified from a previous report^67^, by a BD FACSAria IIIu cell sorter (Becton Dickinson) using a 100-µm sort nozzle (see Supplementary Fig. 1). A sheath pressure of 35 psi and a drop drive frequency of 60 kHz were applied. 43.2 µl of sorted nuclei solution was applied to a Chromium Next GEM Chip without dilution and Chromium Next GEM Single Cell 3’ GEM, Library & Gel Bead Kit (v3.1) was used to generate libraries following the manufacturer’s instructions. Nuclei concentration was monitored by a fluorescent cell counter (Thermo Fisher, Countess 3 FL) using the DAPI channel to obtain approximately 200 nuclei/µl. Libraries were sequenced using a NextSeq (Illumina) machine at high output mode.

For initial wild-type analyses, about 100 hypocotyls from a *PXY*_*pro*_*:H4-GFP;SMXL5*_*pro*_*:H2B-RFP* line collected four weeks after germination were used. Sequencing reads were mapped to the Arabidopsis genome (with H4GFP and H2BRFP transgene sequences added) using STAR (2.7.8a)^68^ with the “--alignIntronMax 10000 --alignMatesGapMax 10000” option. “GeneFull” option was used in STAR solo to include reads mapped to introns. Seurat^69^ (4.0.6, 4.1.0 or 4.3.0) was used for further analysis. Cells with nCount_RNA between 1201 and 9999 and a mitochondrial genome read fraction of less than 20% were kept for further analysis. Clustering and UMAP were generated using default settings of Seurat with the parameters “dims = 1:15, resolution =1.2, algorithm =2”. Monocle 3 (1.3.1)^70^ was used for trajectory analysis and the region around the CSC cluster was selected as the root node for pseudotime calculation.

For wild type and *d14* mutant analysis, about 200 hypocotyls collected 19 days after germination were used. Sequencing reads were mapped to the Arabidopsis nuclear genome with cell ranger (6.0.1, 10x genomics) using the “--include-introns” and “--alignIntronMax 10000 --alignMatesGapMax 10000” alignment options. nCount_RNA between 1501 and 14999 were kept for comparing wild type and *d14* mutants. Clustering was carried out using parameters “dims = 1:15, resolution =1.2, algorithm =2”, and UMAPs were generated after randomly resampling 500 nuclei from each genotype.

For VASA-seq analysis^21^, about 100 hypocotyls from a *PXY*_*pro*_*:H4-GFP;SMXL5*_*pro*_*:H2B-RFP* line were collected four weeks after germination to obtain 1134 ‘cambium region’ nuclei (three 384-well plates). Additional 100 hypocotyls were used for collecting 1134 ‘all region’ nuclei (three 384-well plates) (see Supplementary Fig. 1). Single nuclei were sorted into individual wells of 384-well plates containing well index oligos purchased from Single Cell Discoveries B.V. (Utrecht, The Netherlands), using the index sorting mode of the BD FACSAria IIIu cell sorter, recording the fluorescence signal of each nucleus (Supplementary Data 7). Multi-well plates were frozen and further processed according to the VASA-seq protocol^21^ of Single Cell Discoveries with the following modifications. (1) In the end, repair and polyA reactions, our added mix per well contained 7.5 μM ATP and 3.75 mU of polyA polymerase. (2) We used 6 μl of ExoSAP after in vitro transcription. (3) The composition of our 2.5× RNaseH buffer used during the rRNA depletion step was 125 mM Tris-HCl pH 7.5, 250 mM NaCl, 10 mM MgCl_2_. Sequencing reads were similarly mapped to the Arabidopsis genome (with H4GFP and H2BRFP transgene sequences added) using STAR with the same options mentioned above. Well barcodes were obtained from Gene Expression Omnibus (https://www.ncbi.nlm.nih.gov/geo/; Accession ID: GSE112438, celseq2_bc.csv.gz). Fluorescent signals were combined in Seurat with a 150 offset value to avoid negative values in the 488 nm and 561 nm channels, and all nucleus data were merged. Cells with nCount_RNA values between 1201 and 14,999, a mitochondrial genome read fraction lower than 20%, fluorescent signals in the 405 nm and 488 nm channels lower than 75,000 were kept for further analysis. Clustering and UMAP were performed with parameters “dims = 1:15, resolution =1.8, algorithm =2”.

Basic statistics of all single-cell analyses can be found in Supplementary Data 1. Marker genes for each cluster were generated by using the *FindAllMarkers* function in Seurat (Supplementary Data 2) (only.pos = TRUE, min.pct = 0.25, logfc.threshold = 0.25, test =“wilcox”, return.thresh = 0.01). Seurat object files for each dataset are deposited at Gene Expression Omnibus under accession code GSE224928. Phytohormone responsive genes^24,25^ and marker genes for cell clusters generated by single cell transcriptome analyses^8,9,71^ were curated from previous reports and integrated in Seurat by using the AddModuleScore function (Supplementary Data 3). Differential expression was tested by the Steel-Dwass test using an R script (http://aoki2.si.gunma-u.ac.jp/R/src/Steel-Dwass.R) authored by Shigenobu Aoki (Gunma University, Japan).

### Confocal microscopy

Hypocotyl samples were fixed overnight at 4 °C in  4% (w/v) PFA dissolved in PBS. The tissue was washed twice with PBS, optionally embedded in 5% low gelling temperature agarose, sectioned by razor blades (Wilkinson basic), and then stained with 0.1% DirectRed 23, 0.1% Renaissance SR2200 (Renaissance Chemicals Ltd, UK) or 10 μg/ml Hoechst 33342 for 5 min at room temperature. Excess staining was removed by clearing the sample in 1× PBS. Confocal microscopy experiments were carried out on a Leica TCS SP8 (Leica Microsystems Mannheim, Germany). 458 nm, 514 nm and 561 nm lasers were used to excite mTurquoise2 (CFP), YFP (mVenus), and mCherry/Direct Red, and emissions were detected at 465–509 nm, 524–540 nm and 571–630 nm, respectively. Hoechst 33342 and Calcofluor White, together with lignin in differentiated xylem vessel elements, were visualised using a 405 nm laser, and collection of the emission at 410–450 nm. Basic Fuchsin was visualised using a 561 nm laser by STELLARIS 8 (Leica Microsystems, Mannheim, Germany) or Leica TCS SP8 (Leica Microsystems, Mannheim, Germany), collecting the emission in a range of 586–650 nm. Imaging of promoter reporter lines for cluster annotation validation and in situ hybridisation was carried out on an LSM 710 (Carl Zeiss Microscopy GmbH, Oberkochen, Germany) or on a STELLARIS 8 (Leica Microsystems, Mannheim, Germany). 405 nm, 488 nm and 561 nm lasers were used to excite Renaissance SR2200, GFP, Direct Red 23 and ATTO550, respectively.

### Hybridisation chain reaction (HCR)-based in situ hybridisation

Arabidopsis hypocotyls from four-week-old plants were fixed overnight at 4 °C in 4% (w/v) PFA. After washing off the fixative, sections were produced manually and immediately transferred to 100% methanol for more than 5 min. Hybridisation and amplification were carried out with ISHpalette Short hairpin amplifier ATTO550-S41 (#IPL-R-S41, Nepa Gene Co., Ltd., Ichikawa, Japan) according to the manufacturer’s instructions. See Supplementary Data 5 for oligo sequences used as probes for each gene.

### Strigo-D2 ratio analysis

False colour images were generated using ImageJ by calculating intensity ratios of each pixel from mVenus and mCherry channels after being Gaussian Blurred and subtracting background signals. For calculating the ratio value of each nucleus, nuclear regions were detected by using the Particle Analyzer function in ImageJ after masking the nuclear region through thresholding. Then, nuclear mVenus and mCherry signal intensity were measured and intensity ratios were determined. Nuclei within cambium, phloem, and xylem zones, as well as in developing vessel elements, were manually defined as follows: around six cell layers counting from the vessel element border toward the organ periphery were defined as the cambium zone. Nuclei distal to the cambium were defined as phloem, and the nuclei proximal to the cambium were defined as xylem. The enlarged nuclei within the xylem region were considered to be located in developing vessel elements.

### Histological analyses

The harvested hypocotyls from five week-old (3 weeks SD+2 weeks LD) plants or four week-old plants subjected to two weeks of water deficiency treatment (SD conditions) were infiltrated by 70% ethanol for at least three days at 4 °C before being paraffin embedded by the Leica ASP200 S processor (Leica Microsystems, Mannheim, Germany). After embedding in paraffin, the microtome RM2235 (Leica Microsystems, Mannheim, Germany) was used to produce 10-μm-thick sections. The sections were harvested from the upper part of the hypocotyl, 1 mm below the leaf primordia. Dried sections were deparaffinized, stained with 0.05% toluidine blue (#52040, AppliChem, Darmstadt, Germany) and fixed by Micromount Mounting Media (Leica Microsystems; Mannheim, Germany) on microscope slides (Thermo Scientific; Waltham, USA). Slides were scanned using the Pannoramic SCAN II scanner (3DHistech, Budapest, Hungary) and analysed by the CaseViewer 2.2 software (3DHistech, Budapest, Hungary). Vessel elements were either automatically detected using a developed vessel-counting macro in Fiji or the VesselWizard tool, both established for this purpose^72^ and corrected manually to eliminate false positives or negatives (using the Wand Tool in Fiji).

### Dexamethasone and estradiol treatment

For *SMXL7*_*pro*_*:SMXL7*^*d53*^*-GR* induction by DEX, a stock solution of 25 mM DEX was dissolved in DMSO, and a 15 μM working solution was freshly prepared by diluting the stock solution with tap water. Control treatments contained an equivalent amount of solvent. Plants were initially grown in SD conditions for three weeks without treatment, and treatment was started when plants were transferred to LD conditions by watering twice a week with either 50 ml 15 μM DEX or mock solution per pot until harvest. For estradiol induction of *35S:XVE* > *>miRNA165a* expression^17^, a 20 mM stock solution of 17β-Estradiol (Sigma-Aldrich, E2758) was prepared in DMSO and stored at −20 °C. Five-day-old seedlings were transferred to plates supplemented with either 5 μM 17β-Estradiol or an equal volume of DMSO until they reached two weeks of age. Hypocotyls were harvested and fixed in 4% PFA at 4 °C. Cross-sections, 200 μm thick, were obtained using a Vibratome (Leica VT1000 S) after embedding the samples in low-melting agarose (Sigma-Aldrich, A9414). The sections were stained overnight at 4 °C with 0.0 2% Basic Fuchsin in ClearSee.

### GR24^4DO^ application

2 µM GR24^4DO^ (StrigoLab S.r.l., c/o Dept. of Chemistry, Turin University, Italy) was prepared through a 5000× dilution of a stock solution (10 mM GR24^4DO^ dissolved in acetone). Seedlings were initially grown on plates containing solid 1/2 MS medium with 1% sucrose supplemented for five days (SD conditions) without treatment, subsequently transferred to culture vessels (C1958, PhytoTechLabs, USA) supplemented either with 2 μM GR24^4DO^ or mock solution containing an equivalent amount of acetone, grown in SD condition and harvested four weeks after germination for histological analyses.

### RNAseq and qRT-PCR analysis

#### RNAseq

DEX (Sigma-Aldrich, USA) was dissolved in DMSO as a 25 mM stock solution and stored at −20 °C. The working concentration of DEX was 15 μM diluted from the stock solution by tap water. The DEX-based induction of *SMXL7*_*pro*_*:SMXL7*^*d53*^*-GR* lines was conducted by directly watering 15 μM DEX or an equal volume of DMSO (mock) to four-week-old plants (three weeks SD conditions, then transferred to LD conditions) grown on soil. After five hours of incubation, the hypocotyls from DEX- and mock-treated plants were immediately harvested and ground using a pestle and mortar after being immersed in liquid nitrogen. RNA was isolated using the RNeasy Mini Kit (QIAGEN, Netherlands) and genomic DNA contamination was removed by referring to the protocol of the TURBO DNA-free™ Kit (Thermo Scientific; USA). Subsequent clean-up of RNA was performed by using RNeasy Mini Kit. 1.2 µg of total RNA/sample was used for library preparation using NEBNext Ultra II RNA Library Prep Kit for Illumina (NEB) using the polyA mRNA workflow and Unique Dual Index primers (NEB). 12 cycles of amplification were performed, and the libraries were sequenced using NextSeq 550 (Illumina). Obtained reads were mapped by STAR-2.7.8a with “--alignIntronMax 10000 --alignMatesGapMax 10000 --outFilterMultimapNmax 1” option. Reads per gene were counted by summarizeOverLaps function (GenomicAlignments v1.30.0)^73^ with “mode = “union”, ignore.strand = TRUE” option. Differentially expressed genes were detected using DESeq2 (v1.34.0)^74^ applying a threshold of “baseMean>50, |log2FoldChange|>0.585, padj<0.01”. Functional enrichment analysis was performed using g:GOSt (version: e111_eg58_p18_f463989d)^75^.

#### qRT-PCR

Hypocotyls of four-week-old wild-type and *d14* mutant plants grown on soil were harvested. Total RNA was extracted using the method mentioned above. cDNA synthesis was performed according to the instructions of the Thermo Revert Aid Kit (Thermo Scientific; Waltham, USA). Real-time PCR assays were conducted using SYBR Green Mix (Thermo Scientific; Waltham, USA) and gene-specific primers (Supplementary Data 5) on a qTOWER3 thermal cycler and using the *EF1-a* (*AT5G60390*) gene as an internal reference.

### Water deficiency treatments

To grow plants under comparable conditions, each pot contained 70 g of soil. For watering, pots were placed in petri dishes to soak 25 ml of water overnight from below. Next, 20 ml of nematode-containing solution was added to each pot from above. For pot weight measurement, pots were kept in petri dishes during subsequent watering (20 ml every week during the first three weeks and 25 ml twice a week for the last two weeks). Pot weight was measured starting five weeks after germination for the consecutive 12 days. For histological analysis of stress-related phenotypes, plants were grown well watered for two weeks (20 ml every week), before water deficiency treatment was applied for two weeks. The control group was kept in well-watered conditions during the treatment.

### Stomatal conductance measurements

Wild type, *d14*, *max2* and *smxl6;7;8* plants were grown in growth chambers with sufficient watering for four weeks, followed by water deficiency treatments for 12 days. During the first three weeks, plants were watered with 20 ml every week during the first three weeks and 30 ml twice a week for the next three weeks. DEX or mock-treated *PXY*_*pro*_*:GR-MPΔIII/IV* plants were not watered for 10 days after the DEX or DMSO treatment. Stomatal conductance of wild type, *d14*, *max2* and *smxl6;7;8* plants was measured after 12 days of water deficiency treatment and DEX or mock-treated *PXY*_*pro*_*:GR-MPΔIII/IV* plants were analysed after 10 days of water deficiency treatment. For the measurements, the SC-1 Leaf Porometer (METER Environment®, Meter Group, Pullman, US) was clipped on the abaxial side of the leaf. The 9th, 10th and 11th produced leaves of wild type, *smxl6;7;8*, DEX or mock-treated *PXY*_*pro*_*:GR-MPΔIII/IV* plants and three well expanded upper leaves from *d14* or *max2* mutants were chosen for the measurement. The SC-1 Leaf Porometer was used according to the user manual. Single measurements were temporarily randomised across the course of the day and across genotypes.

### Stomata imprints

Water deficiency-treated plants used for the stomatal conductance measurement were taken to imprint the abaxial leaf side for stomata density quantification (stomata/mm^2^). For the imprint, a small drop of instant adhesive glue (UHU Sekundenkleber blitzschnell; UHU, Bühl, Germany) was placed on a Superfrost™ Microscope slide, and the leaf was gently pressed on the glue for two seconds. The imprints were visualised using a Contrast Microscope DMIRB microscope with a 20× objective and the bright field mode. Five images of the central leaf regions of each imprint were taken. The number of stomata was counted using Fiji.

### CFDA application

Roots of three-week-old plants (grown on plates) were cut 2 cm below the hypocotyl, and 5 µl of 1 mM CFDA (5(6)-Carboxyfluorescein diacetate N-succinimidyl ester, Sigma-Aldrich, USA) or DMSO were applied. After 30 min, hypocotyls were harvested, fixed with 4% PFA and embedded in 9% low-gelling temperature agarose. 100 µm thick vibratome sections were used for confocal microscopy.

### Basic Fuchsin staining

To observe xylem strands in roots, five-day-old seedlings were stained and fixed in 0.2% (m/v) Basic Fuchsin dissolved in ClearSee^76,77^ (10% xylitol, 15% sodium deoxycholate, and 25% urea) solution overnight. Next, the fuchsin solution was removed, and samples were washed once with ClearSee for 30 min. Subsequently, seedlings were stored in ClearSee solution and analysed.

### Statistics and reproducibility

All the measurements were done on distinct samples. Statistical tests were applied in a two-sided mode. Box plots indicate the 25th (Q1, box limit), 50th (median, centre line) and 75th (Q3, box limit) percentiles and whiskers indicate the value range or up to the 1.5× interquartile range from the Q1 or Q3 limit, respectively. Data points beyond this range were plotted individually as outliers. Statistical analyses were carried out using R (v4.0.4 or v4.0.5), ggplot2 (v3.3.3), or Python (v3.10.7), pandas (v1.5.0), rstatix (v0.7.2) and seaborne (v0.12.0).

No statistical methods were used for sample-size calculation. Sample sizes have been maximised according to practical considerations. No data were excluded from the analyses except in snRNA-seq analysis, nuclei with a low number of molecules detected were excluded from further analysis according to the standard pre-established analysis pipeline. All the findings were confirmed by usually three but at least two replicates. snRNA-seq analyses were not replicated using the exact same method and type of samples, however, findings were confirmed in each analysis using different technologies and types of samples, suggesting their universality. Plant pot positions were randomised in possible cases, however, randomisation was not applied to all the experiments due to practical reasons (enhanced physical handling of plants). Covariates were controlled by applying the exact same conditions (growth substrate, temperature, light) to all individuals. The investigators were not blinded to allocation during experiments and outcome assessment.

### Reporting summary

Further information on research design is available in the Nature Portfolio Reporting Summary linked to this article.

## Supplementary information

Supplementary information

Description of Additional Supplementary Files

Supplementary Data 1

Supplementary Data 2

Supplementary Data 3

Supplementary Data 4

Supplementary Data 5

Supplementary Data 6

Supplementary Data 7

Reporting Summary

Transparent Peer Review file

## Source data

Source Data

## Data Availability

The raw sequencing data of snRNA-seq and bulk RNA-seq analyses, and Seurat object files of snRNA-seq data generated in this study have been deposited at NCBI’s Gene Expression Omnibus database under accession code GSE224928 or GSE270808. The authors declare that all other data supporting the findings of this study are mentioned in the main text or the supplementary materials. Source data are provided with this paper.

## Code availability

General codes used for analyses and specific parameters are provided in the “Methods” section. Codes for the presented vessel identification tools are deposited at GitHub [https://github.com/thomasgreb/Zhao-et-al_SL-vessels]^72^.
