# Supplementary material for: Strigolactones optimise plant water usage by modulating vessel formation
Source: Nat Commun. 2025 Apr 28;16:3854. doi: 10.1038/s41467-025-59072-y (PMC12037892; doi:10.1038/s41467-025-59072-y)
Supplement: Supplementary file 1 — Supplementary information [file 41467_2025_59072_MOESM1_ESM.pdf]

Supplementary Information for

Strigolactones optimise plant water usage by modulating vessel formation

Jiao Zhao[1,*], Dongbo Shi[1,2,3,*,✉], Kiara Kaeufer[1*], Changzheng Song[1], Dominik Both[1], Anna Lea Thier[1], Hui Cao[2], Linus Lassen[2], Xiaocai Xu[4], Yuki Hamamura[5], Laura Luzzietti[1], Tom Bennett[6], Kerstin Kaufmann[4], Thomas Greb[1,✉]

Affiliations:

[1]Developmental Physiology, Centre for Organismal Studies, Heidelberg University, Heidelberg, Germany

[2]Genetics, Institute of Biochemistry and Biology, University of Potsdam, Potsdam, Germany

[3]RIKEN Center for Sustainable Resource Science, Yokohama, Japan

[4]Plant Cell and Molecular Biology, Institute of Biology, Humboldt-Universität zu Berlin, Berlin, Germany

[5]Max Planck Institute of Molecular Plant Physiology, Potsdam, Germany

[6]School of Biology, Faculty of Biological Sciences, University of Leeds, Leeds, UK

✉correspondence: tohaku.seki@riken.jp, thomas.greb@cos.uni-heidelberg.de

*These authors contributed equally to this work.

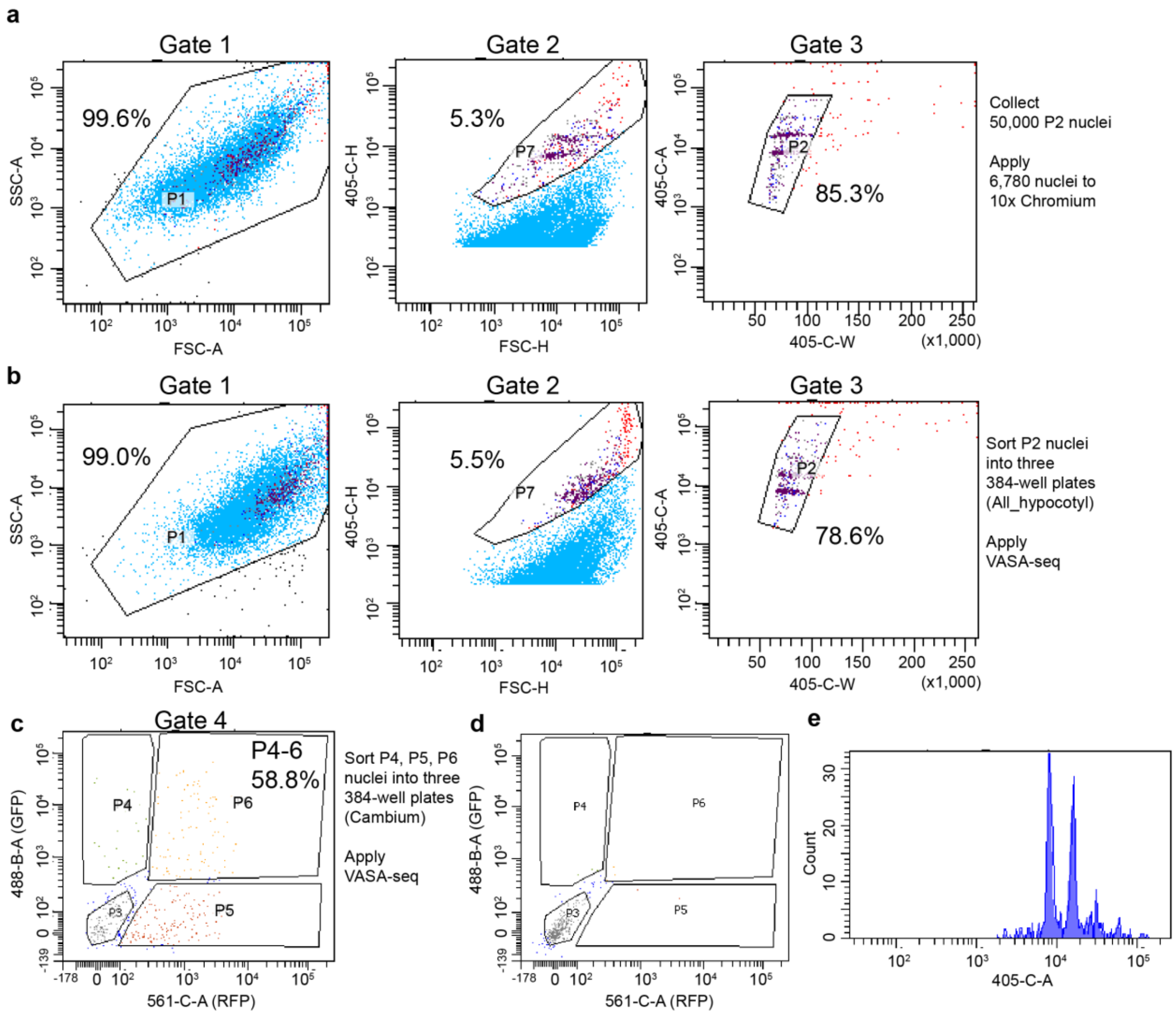

Supplementary Fig. 1: FACS gate settings for nucleus purification.

a, Gate setting and dot plot of the FACSAria cell sorter for nucleus purification and 10x Chromium application. Nuclei were purified through Gate P1, P7 and P2 in a sequential manner. The percentages of each population compared to the parent population are indicated. FSC: forward scatter; SSC: side scatter; 405-C: fluorescence excited by 405 nm laser (hoechst nucleus staining); -A: area; -H: hight; -W: width. **b–c**, Gate setting and dot plot of the FACSAria cell sorter for nucleus purification and VASA-seq application. Nuclei were purified through Gate P1, P7 and P2 in a sequential manner. Individual P2 nuclei were sorted into single wells of three 384-well plates as 'nuclei from the whole hypocotyl' (**b**). (**c**) P2 nuclei were further gated by fluorescence intensity, and individual GFP-positive/RFP-negative (P4), GFP-negative/RFP-positive (P5), or GFP-positive/RFP-positive (P6), nuclei were collected into single well of three 384-well plates as 'cambium nuclei'. *PXY*$_{pro}$*:H4-GFP;SMXL5*$_{pro}$*:H2B-RFP* plants were prepared independently for (**b**) and (**c**). **d,** Dot plot of the FACSAria cell sorter obtained from wild type plants using the same gate settings as used in (**c**) as a control. **e,** Histogram of hoechst signals of P2 nuclei demonstrating nucleus integrity.

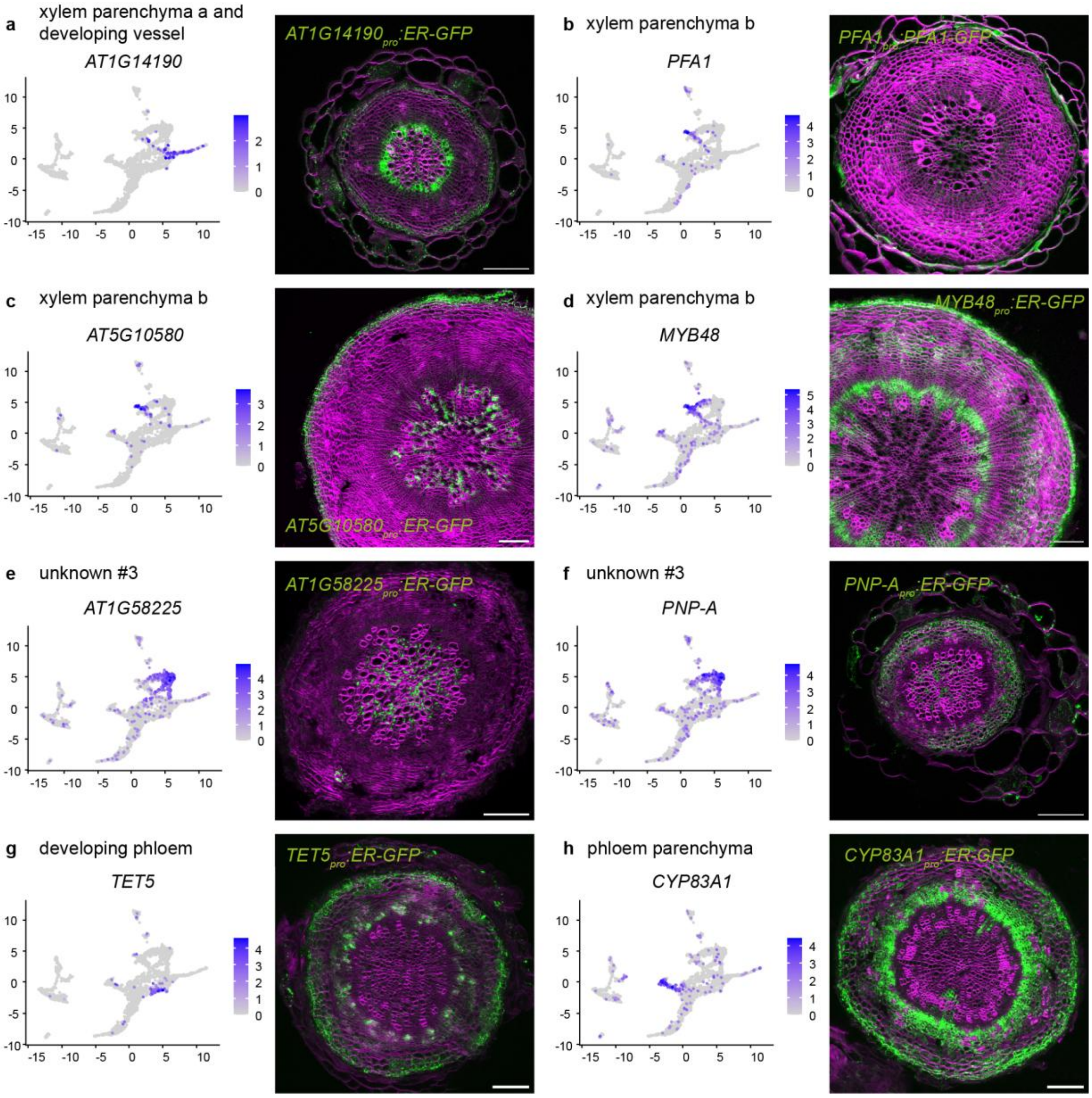

Supplementary Fig. 2: Activity of transcriptional or translational reporters for vascular cluster-specific genes identified in the 10x snRNA-seq dataset.

a–**h**, Transcript abundance in the UMAP plot (left; see Fig. 1 for annotation) and maximum intensity projection of confocal images obtained from hypocotyl cross-sections of plants carrying promoter reporter transgenes (right): Xylem parenchyma a and developing vessel cluster (**a**, *AT1G14190*), xylem parenchyma b cluster (**b**, *PERICYCLE FACTOR TYPE-A* (*PFA)1/AT1G31050*, **c**, *AT5G10580,* **d**, *MYB48/AT3G46130*), unknown #3 cluster (**e**, *AT1G58225,* **f**, *PLANT NATRIURETIC PEPTIDE A (PNP-A)/AT2G18660*), developing phloem cluster (**g**, *TETRASPANIN* (*TET*)*5/AT4G23410*), phloem parenchyma (**h**, *CYTOCHROME P450, FAMILY 83, SUBFAMILY A* (*CYP83A*)*1/AT4G13770*). GFP signals are shown in green. Cell walls were stained by Direct Red 23 or Renaissance SR2200 and are shown in magenta. Scale bars represent 100 µm.

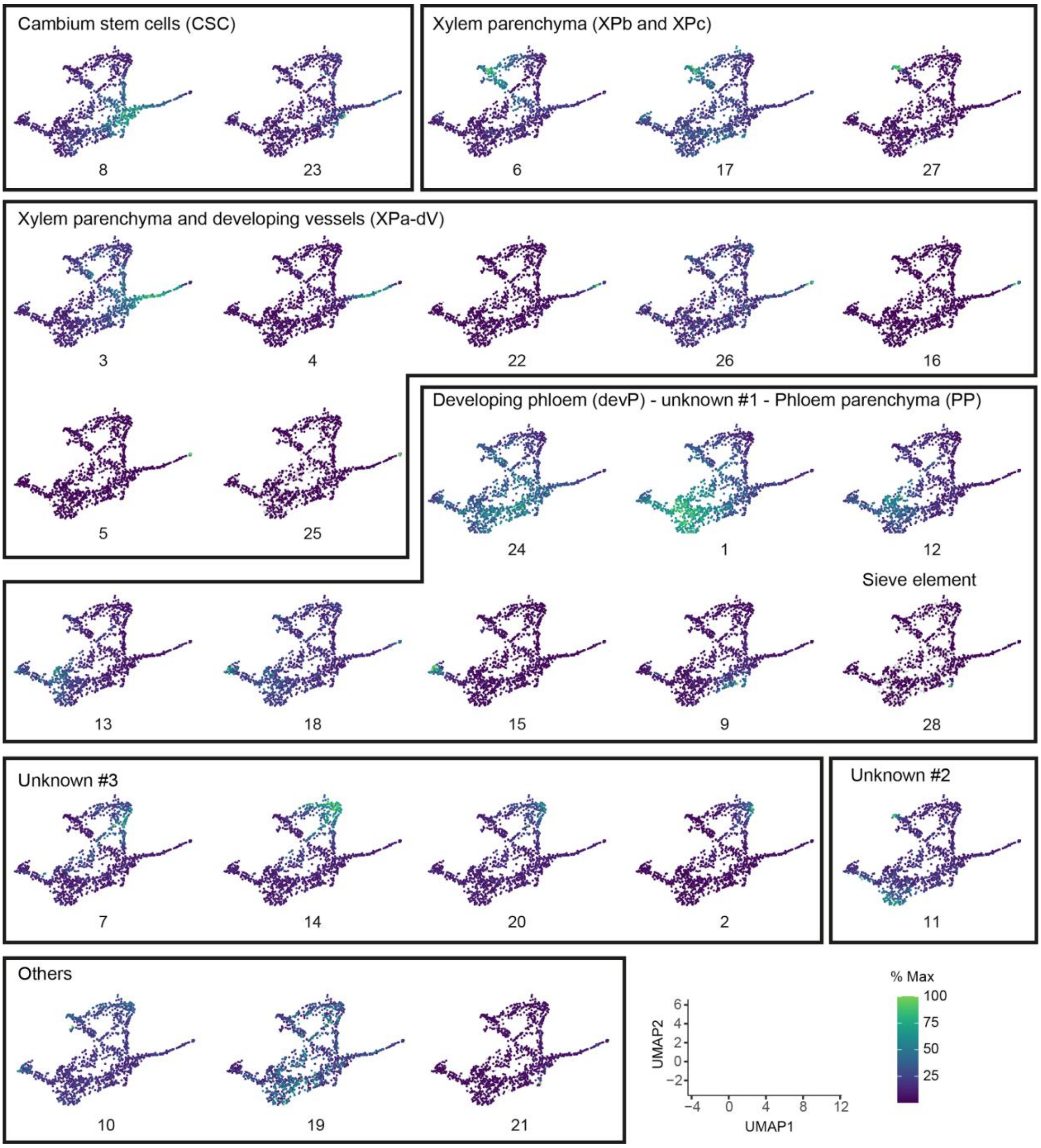

Supplementary Fig. 3: Detailed gene clustering analysis based on the 10x snRNA-seq dataset taking the pseudotime trajectory analysis into account.
Genes were categorised in 28 modules based on the detected expression patterns in cambium-related cells. The aggregate transcript abundance of all the genes categorised in each gene module is shown in each UMAP plot and the ID number of the gene module is labelled at the bottom of each UMAP plot. Transcript abundance is shown in a colour-scale by the percentage of the abundance in the cell with the highest value. See Fig. 1 for UMAP annotation. See Supplementary Data 2 for gene lists of each module.

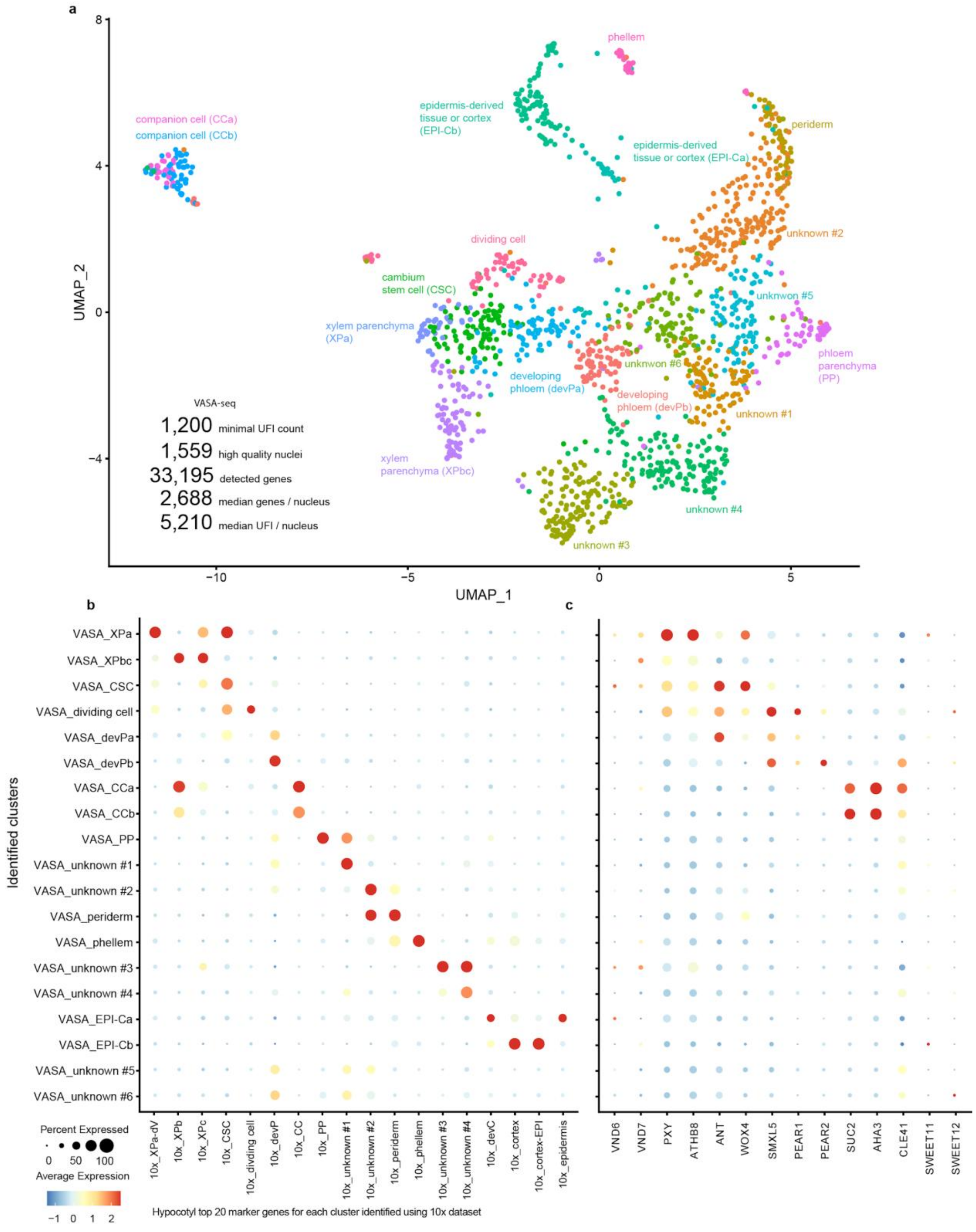

Supplementary Fig. 4: Identification of hypocotyl cell types using VASA-seq.
a, UMAP plot of VASA-seq analysis using 1,559 hypocotyl nuclei organised in 19 clusters obtained through unsupervised clustering. **b**–**c**, Dot plots showing the expression of tissue-specific genes identified by 10x Chromium analysis (Supplementary Data 1, 2) (**b**) and previously characterised tissue-specific marker genes (**c**), validating the annotation of cluster identities. The size of circles represents the percentage of cells with expression (percent expressed), whereas the colour indicates the scaled average expression (average expression). XP: xylem parenchyma; CSC: cambium stem cells; devP: developing phloem; CC: companion

cells; PP: phloem parenchyma; EPI-C: epidermis-cortex. Source data are provided as a Source Data file.

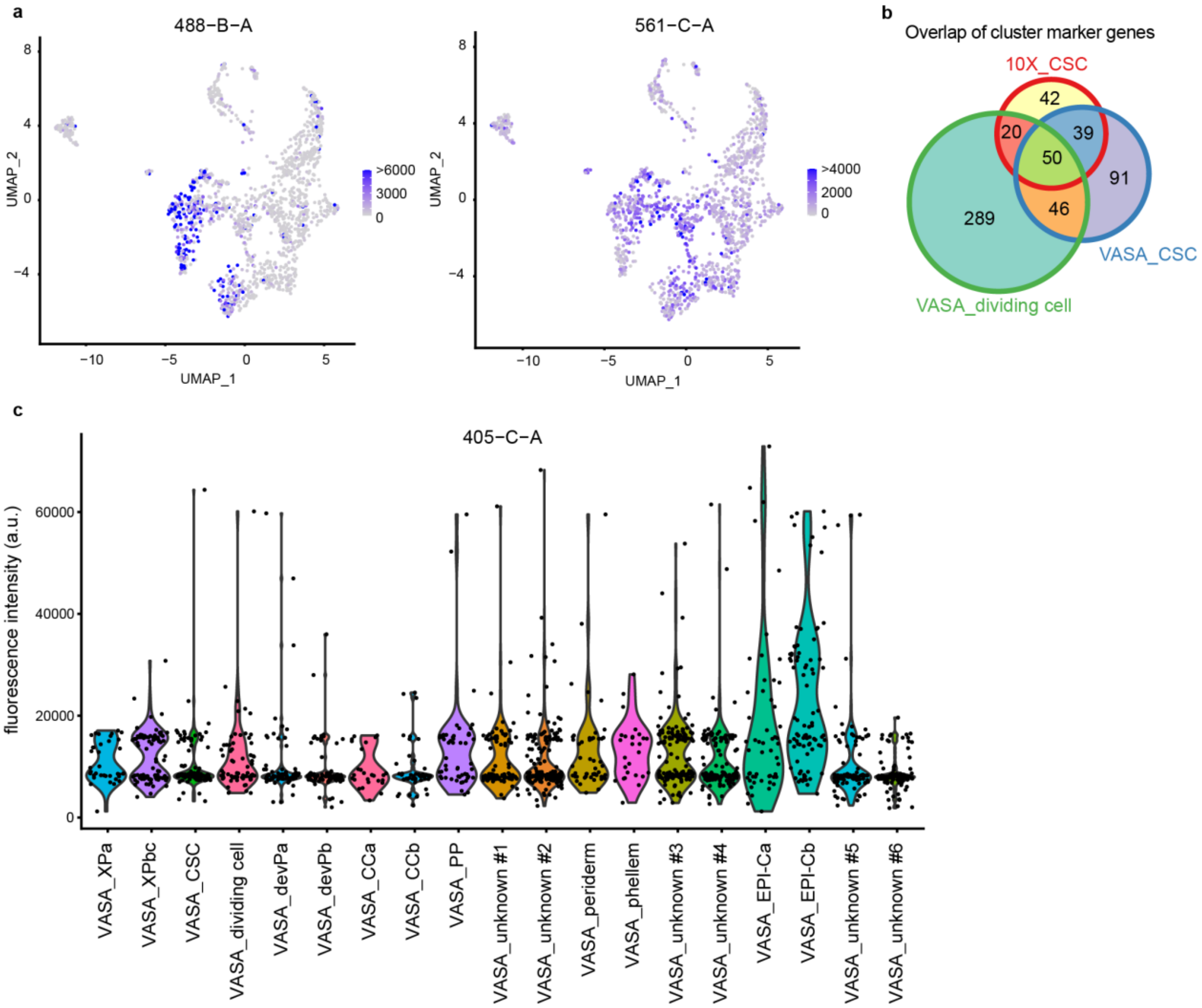

Supplementary Fig. 5: Nucleus fluorescence detected during VASA-seq analyses and CSC-specific marker genes.

a, UMAP plots showing the fluorescence intensity of nuclei collected from *PXY*$_{pro}$*:H4-GFP*;*SMXL5*$_{pro}$*:H2B-RFP* plants captured during the sorting and excited by 488 nm (GFP, left) or 561 nm (RFP, right) laser light, respectively. **b**, Venn diagram showing the overlap of cluster-specific marker genes identified in the 10X_CSC, VASA_CSC and VASA_dividing cell clusters. **c**, Violin plot showing the Hoechst nuclear fluorescence signal excited by 405 nm laser light for each nucleus cluster. Source data are provided as a Source Data file.

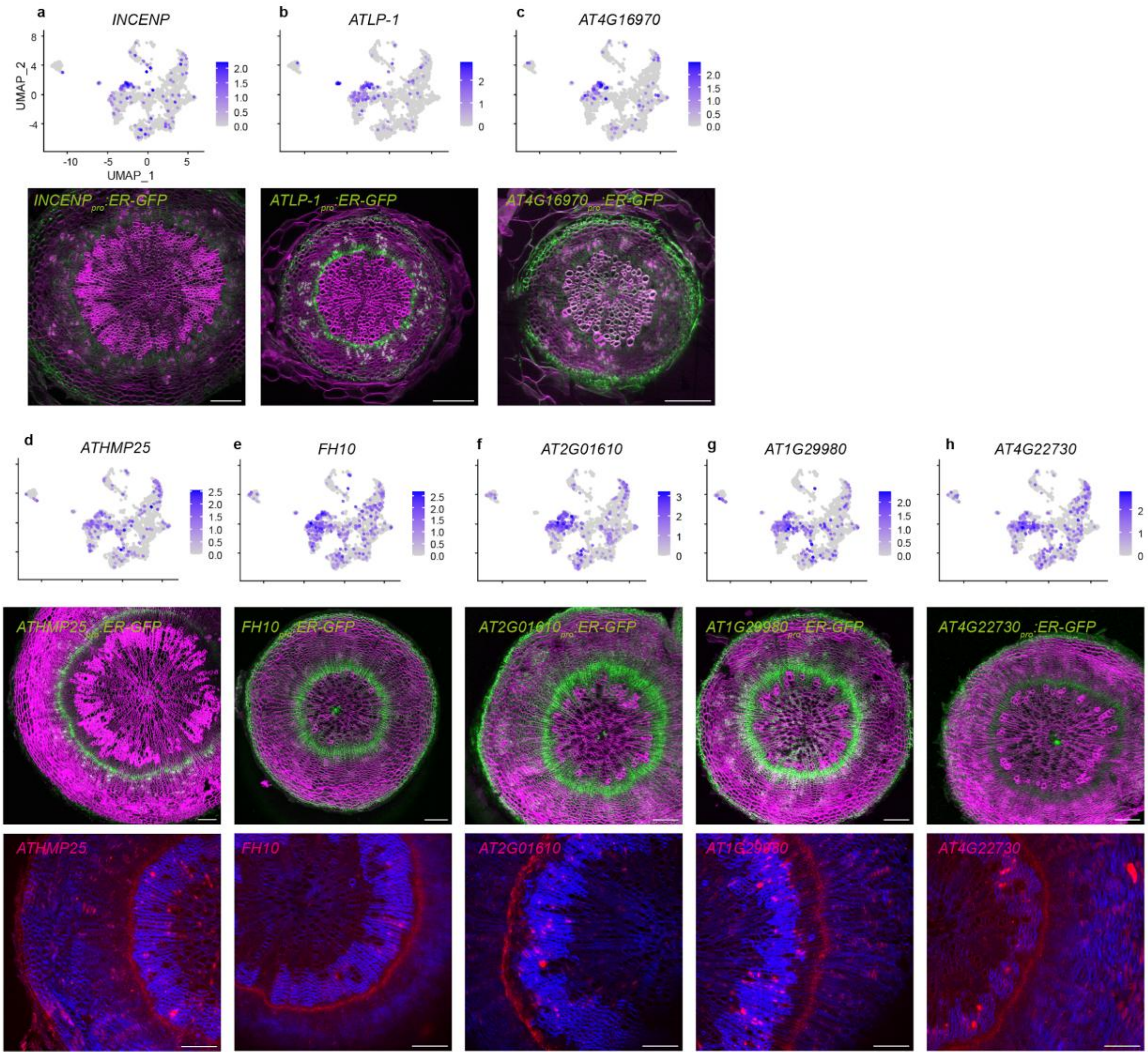

Supplementary Fig. 6: Activity of transcriptional reporters and hybridization chain reaction (HCR)-based *in situ* hybridization for cambium-related genes identified in 10x and VASA snRNA-seq dataset.

a–**h**, Transcript abundance in the UMAP plot of the VASA-seq dataset (top; see Supplementary Fig. 4 for annotation) and maximum intensity projection of confocal images (bottoms) obtained from hypocotyl cross-sections of plants carrying promoter reporter transgenes (GFP, shown in green) and/or *in situ* hybridization (shown in red) of each gene listed below. **a**, *INNER CENTROMERE PROTEIN (INCENP)/AT5G55820*; **b**, *THAUMATIN-LIKE PROTEIN (ATLP)1/AT1G18250*; **c**, *AT4G16970*; **d**, *HEAVY METAL ASSOCIATED PROTEIN (ATHMP)25/ AT3G06130*; **e**, *FORMIN HOMOLOGY (FH)2/AT3G07540*; **f**, *AT2G01610*; **g**, *AT1G29980*; **h**, *AT4G22730*. Cell walls were stained by Direct Red 23 and Renaissance SR2200 and are shown in magenta and blue, respectively. Scale bars represent 100 µm.

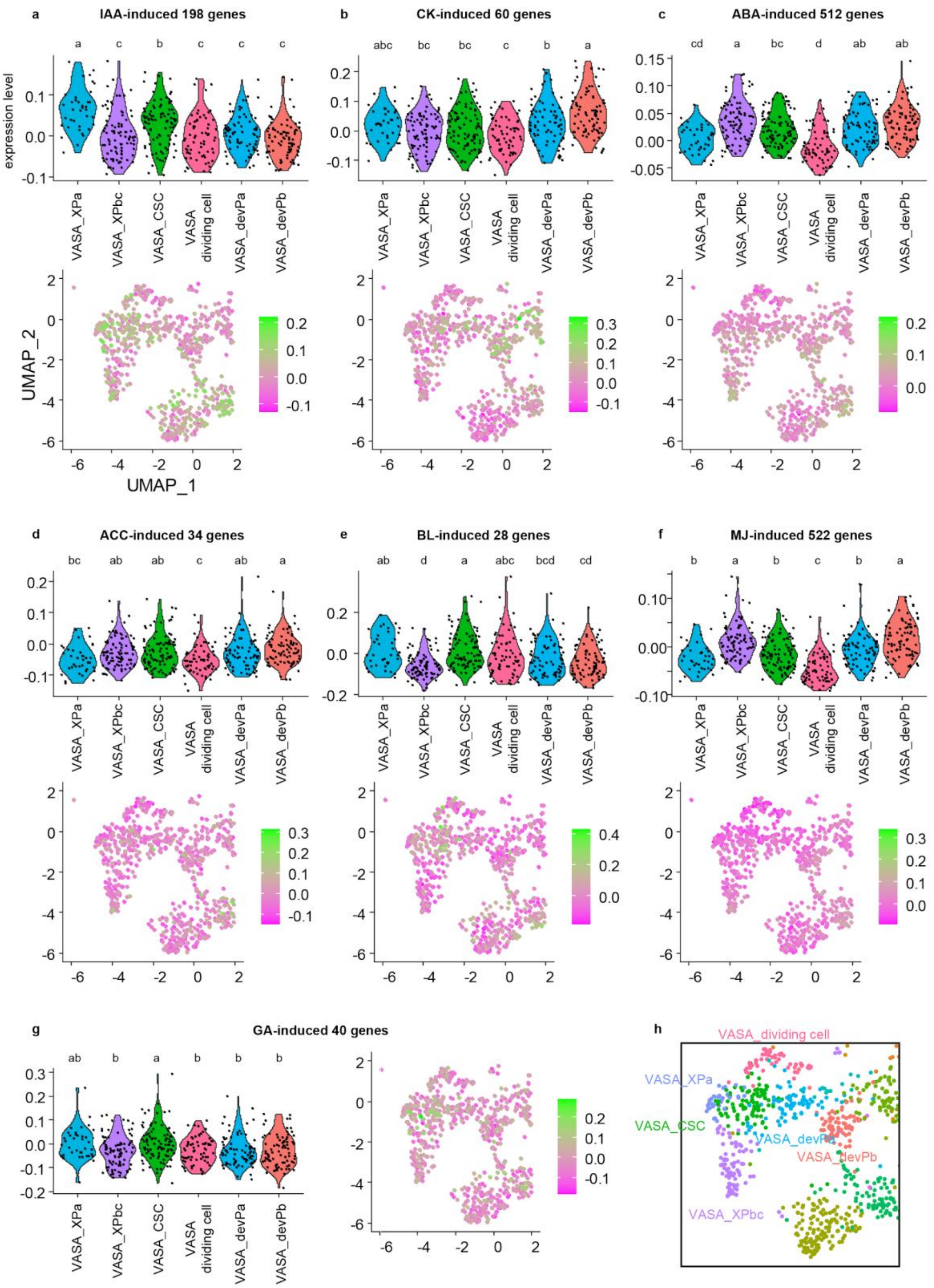

Supplementary Fig. 7: Expression of phytohormone-inducible genes in cambium-related clusters identified in the VASA-seq analyses.

a–**g**, Violin plot (top) and UMAP visualisation (bottom) of transcript abundance of 198 indole 3 acetic acid (IAA; auxin)-inducible genes (**a**), 60 zeatin (CK; cytokinin)-inducible genes (**b**), 512 abscisic acid (ABA)-inducible genes (**c**), 34 1-amino-cyclopropane-1-carboxylic acid (ACC; ethylene precursor)-inducible genes (**d**), 28 brassinolide (BL; brassinosteroid)-

inducible genes, 522 brassinolide (BL; brassinosteroid)-inducible genes (**e**), methyl jasmonate (MJ; jasmonate)-inducible genes (**f**) and 40 gibberellic acid 3 (GA)-inducible genes (**g**) curated from Nemhauser et al., 2006[24] in the cambium-related cell clusters. The result of the Steel-Dwass test for multiple comparisons is indicated by letters ($p < 0.05$). Gene lists used in this analysis can be found in Supplementary Data 3. **g**, A close-up of the UMAP generated during VASA-seq cluster identification (Supplementary Fig. 4) is shown as a reference. Source data are provided as a Source Data file.

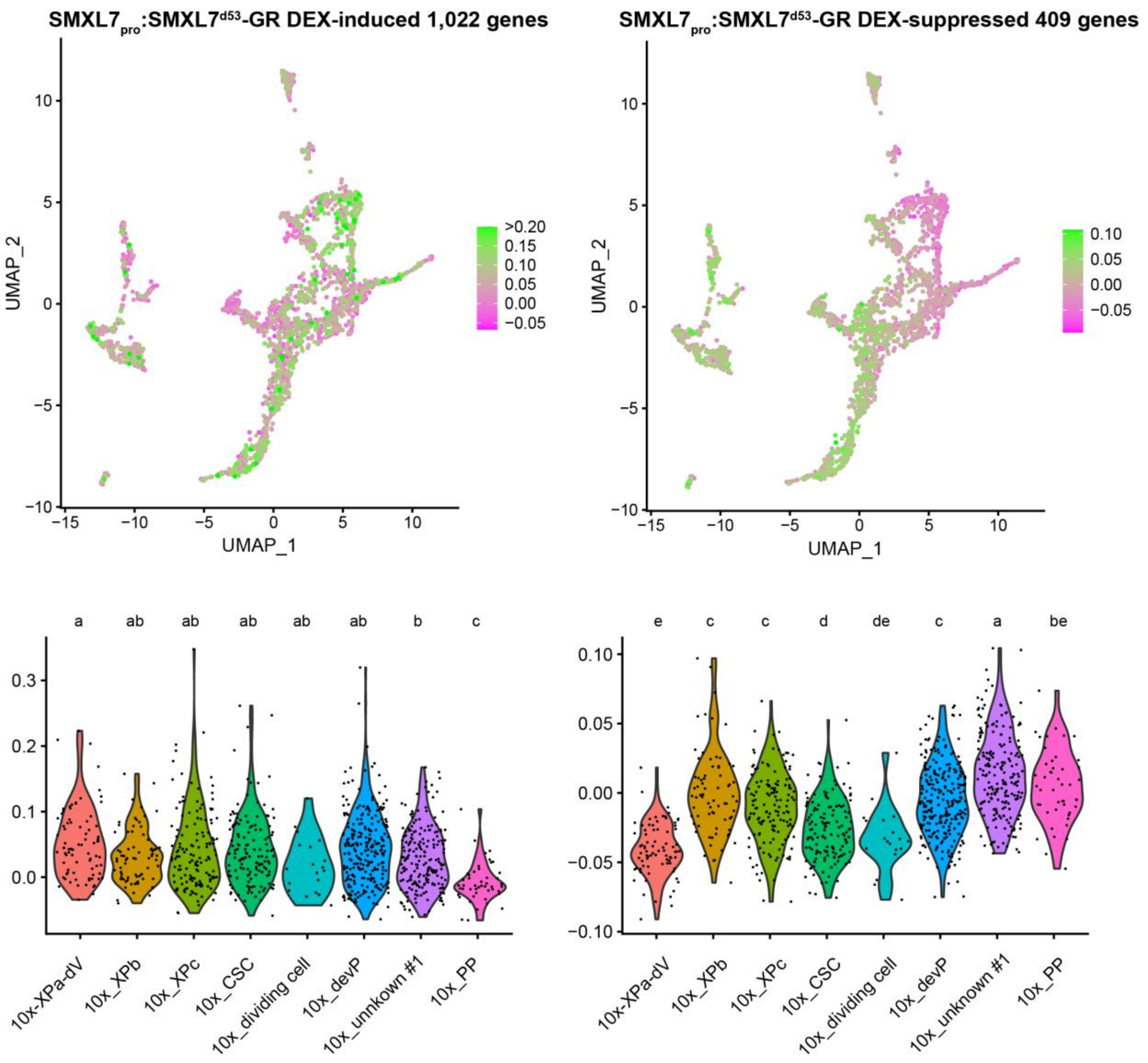

Supplementary Fig. 8: Expression of *$SMXL7_{pro}$:$SMXL7^{d53}$-GR* DEX-induced genes and $GR24^{4DO}$-suppressed genes in 10x snRNA-seq dataset.

Transcript abundance of *$SMXL7_{pro}$:$SMXL7^{d53}$-GR* DEX-induced genes and $GR24^{4DO}$-suppressed genes is shown in UMAP visualisation (top) and violin plot (bottom). Statistical groups determined by the Steel-Dwass test for multiple comparisons are indicated by letters ($p < 0.05$). Source data are provided as a Source Data file.

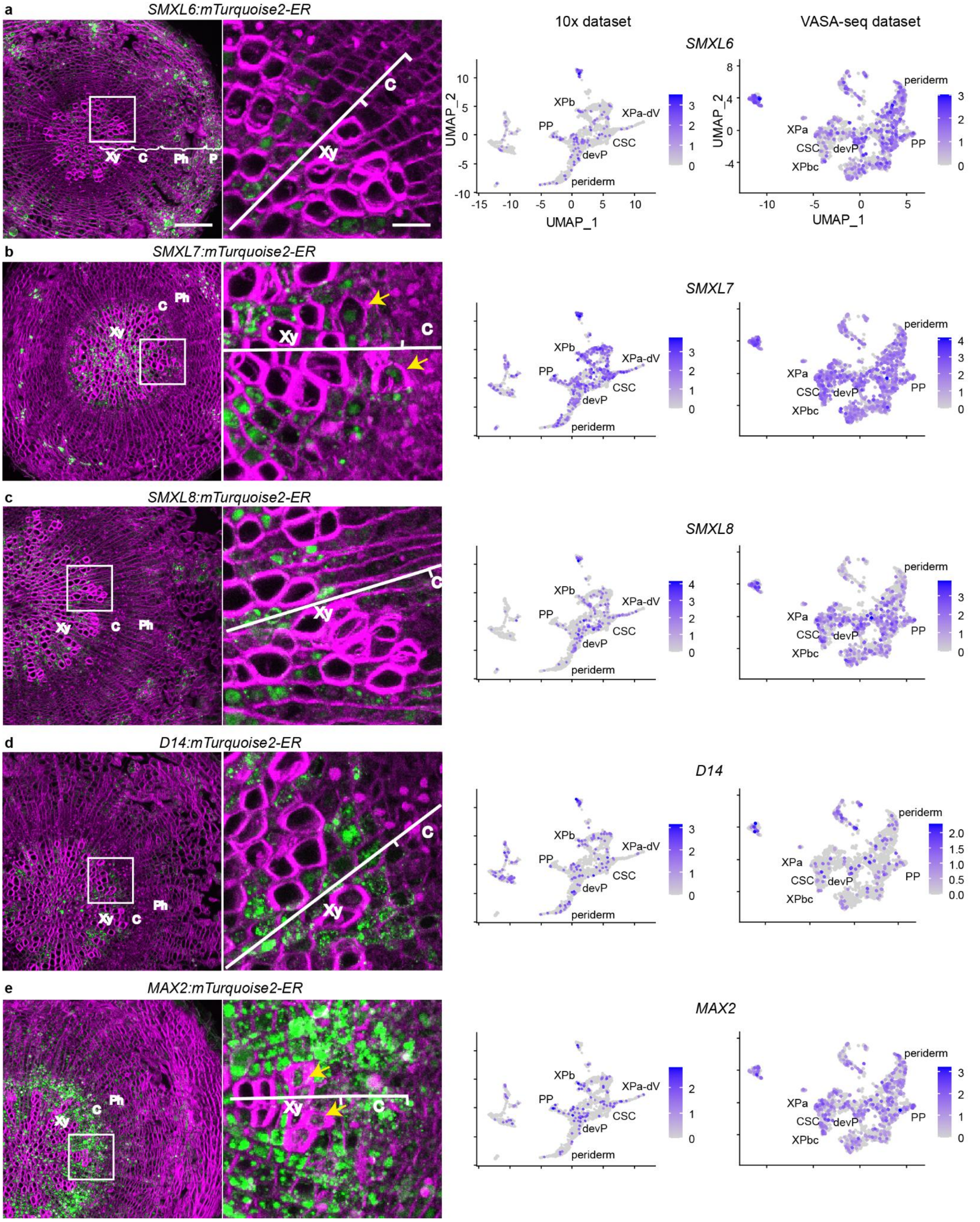

Supplementary Fig. 9: Activity of transcriptional reporters for genes involved in SL signalling in hypocotyl sections and expression patterns detected in snRNA-seq datasets. **a**–**e**, Maximum intensity projection of confocal images obtained from hypocotyl cross-sections of four week-old plants carrying *SMXL6*$_{pro}$*:mTurquoise2-ER* (**a**), *SMXL7*$_{pro}$*:mTurquoise2-ER* (**b**), *SMXL8*$_{pro}$*:mTurquoise2-ER* (**c**), *D14*$_{pro}$*:mTurquoise2-ER* (**d**), or *MAX2*$_{pro}$*:mTurquoise2-ER* (**e**) transgenes, respectively (left). mTurquoise2 signals are shown in green. Cell walls were stained by Direct Red 23 and are shown in magenta. Magnified images of white squared regions are shown on the right of each figure. Yellow arrows indicate developing vessel elements. Scale

bars represent 100 µm on the left images, and 20 µm in the magnified images on the right. White lines indicate the corresponding domains of cambium and xylem. Xy: xylem; C: cambium; Ph: Phloem; P: Periderm. Transcript abundance of each gene in the UMAP plot of 10x and VASA-seq datasets are shown on the right, respectively. Abbreviations for annotated clusters are XP: xylem parenchyma; CSC: cambium stem cell; devP: developing phloem; PP: phloem parenchyma. See Fig. 1 and Supplementary Fig. 4 for detailed annotation.

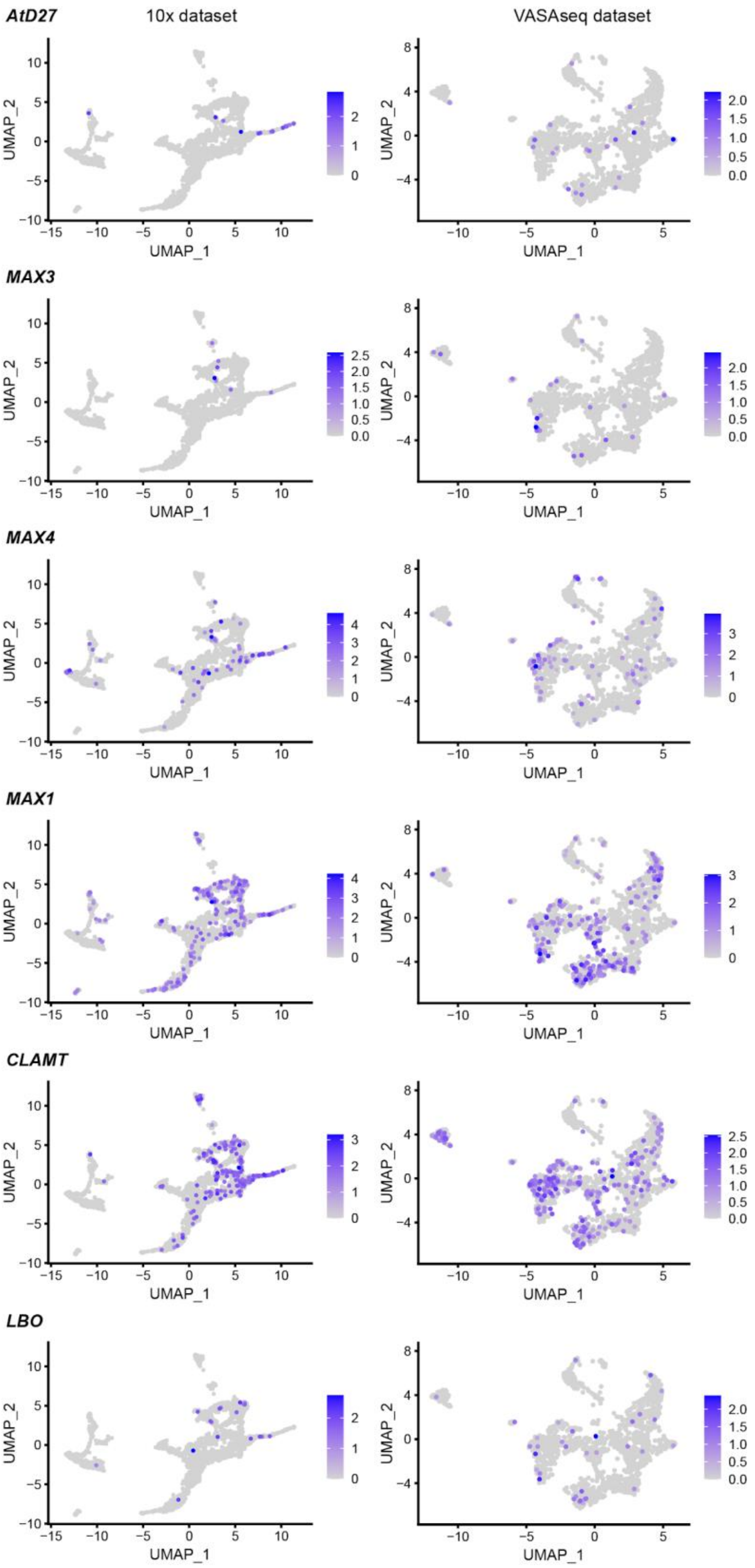

Supplementary Fig. 10: Expression patterns of SL biosynthetic genes detected in snRNA-seq datasets.

Transcript abundance of each SL biosynthetic gene in the UMAP plot of 10x and VASA-seq dataset are shown on the right, respectively. *AtD27*: Arabidopsis *DWARF27* (*AT1G03055*); *MAX3* (*AT2G44990*); *MAX4* (*AT4G32810*); *MAX1* (*AT2G26170*); *CLAMT*: Carlactonoic Acid Methyltransferase (*AT4G36470*); *LBO*: Lateral Branching Oxidoreductase (*AT3G21420*). See Fig. 1 and Supplementary Fig. 4 for detailed annotation.

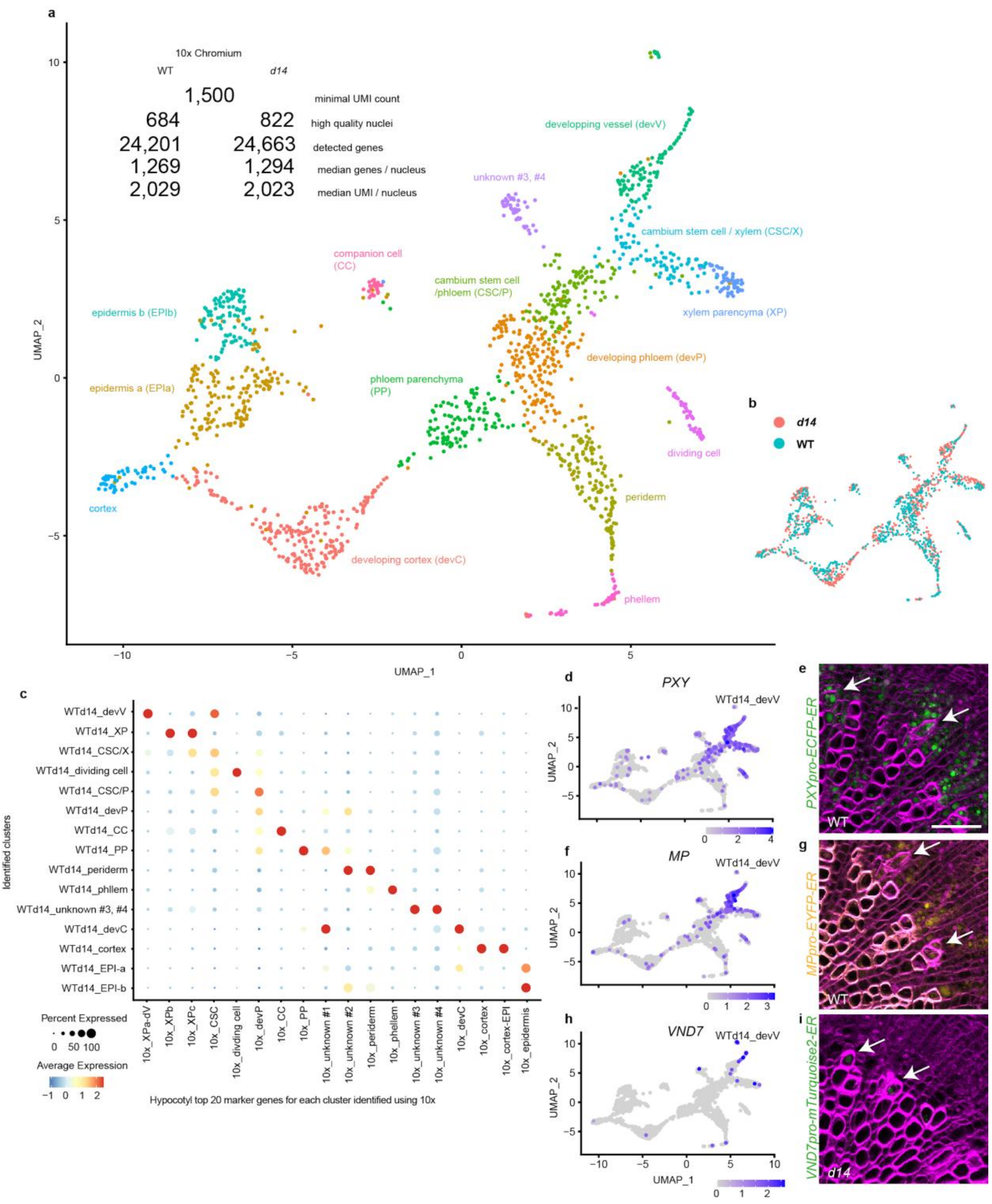

Supplementary Fig. 11: Identification of hypocotyl cell types applying 10x Chromium on wild type and *d14* mutants.

a, UMAP plot of snRNA-seq analysis using in total 1,506 nuclei collected from wild type and *d14* mutants organised in 15 clusters obtained through unsupervised clustering. **b**, UMAP plot colour-coded by genotype. **c**, Dot plot showing the expression of tissue-specific genes identified by 10x Chromium analysis (Supplementary Data 2,, Supplementary Data 3). The size of the circles represents the percentage of cells with expression (percent expressed), whereas the colour indicates the scaled average expression (average expression). Abbreviations for annotated clusters are devV: developing vessel; XP: xylem parenchyma; CSC/X: cambium stem cell/xylem; CSC/P: cambium stem cell/phloem; devP: developing phloem; CC:

companion cell; PP: phloem parenchyma; devC: developing cortex; EPI: epidermis. **d, f, h**, Transcript abundance of the *PXY*, *MP* and *VND7* genes in the UMAP plot shown in (**a**). **e, g, i**, Hypocotyl cross-sections from plants carrying *PXY$_{pro}$:ECFP-ER* (**e**), *MP$_{pro}$:EYFP-ER* (**g**) or *VND7$_{pro}$:mTurqouise2-ER* transgenes (e, g: wild type; i: *d14*) (**i**). Arrows indicate developing vessel elements. Scale bar represents 50 µm. Source data are provided as a Source Data file.

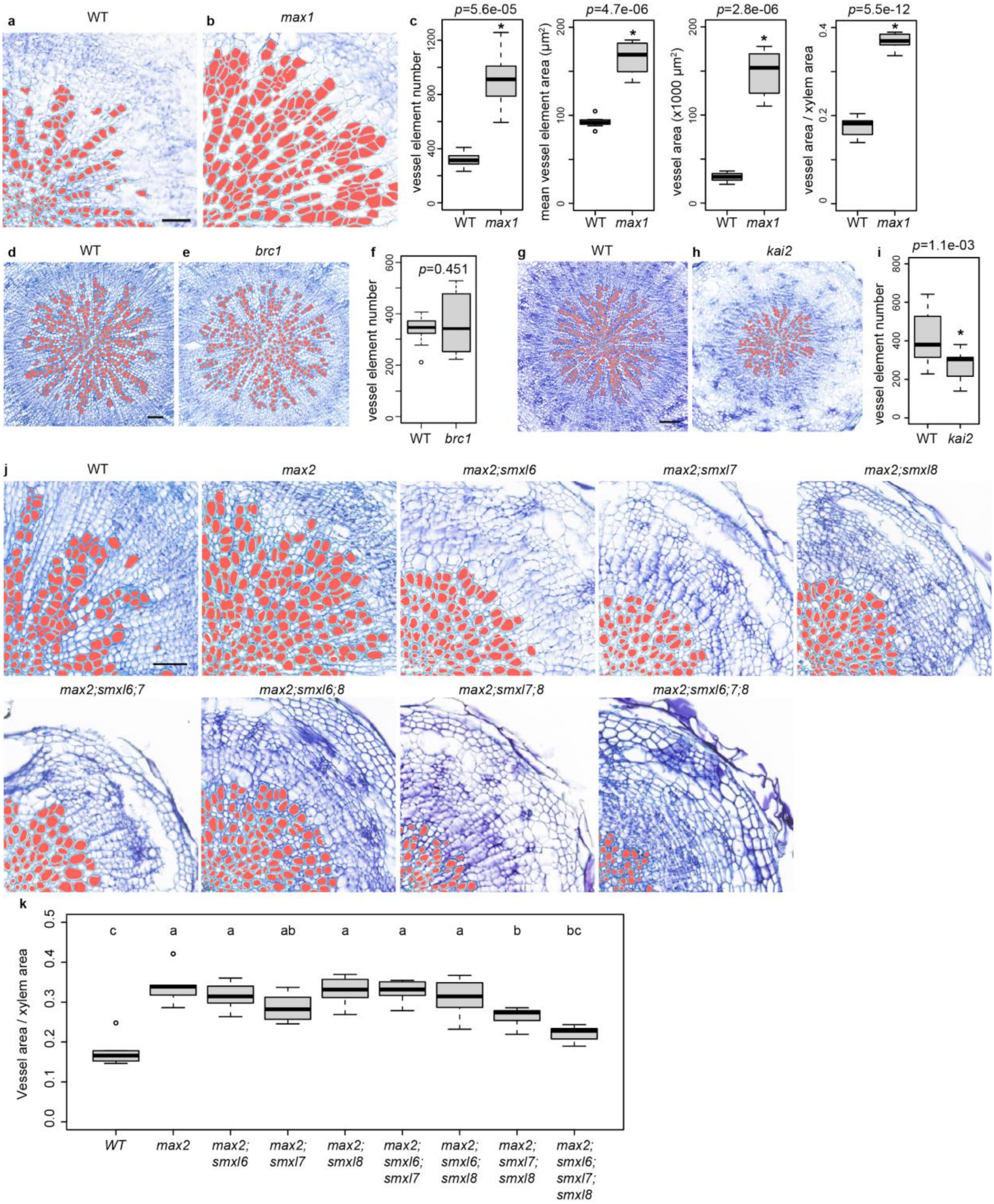

Supplementary Fig. 12: Histological analysis of *max1*, *brc1* mutants and higher order of *max2*, *smxl6*, *smxl7* and *smxl8* mutants.

a–**b**, Toluidine blue-stained hypocotyl cross-sections from five week-old wild type (**a**) and *max1* (**b**) mutant plants. **c**, Quantification of the vessel element numbers per section, the average area of individual vessel elements, the total vessel area per section and the ratio between the vessel element area and the total xylem area in different genotypes. n=9 (wild type) and n=8 (*max1*). **d**, **e**, Toluidine blue-stained hypocotyl cross-sections from five week-old wild type (**d**) and *brc1* (**e**) plants. **f**, Quantification of vessel elements per section comparing wild type and *brc1* plants. n=11 (wild type) and 10 (*brc1*). *p* value was determined by the two-sided Welch's t-test (**c**, **f**) and asterisks indicate $p<0.01$. **g, h,** Toluidine blue-stained hypocotyl

cross-sections from five week-old wild type (**g**) and *kai2* (**h**) plants. i, Quantification of vessel elements per section comparing wild type and *kai2* plants. n=15 for each genotype. *p* value was determined by the two-sided Welch's t-test. This assay was conducted in parallel with the Fig. 3e vessel element analysis, and both share the same wild type data. **j**, Toluidine blue-stained hypocotyl cross-sections from five week-old wild type (WT), *max2* and higher order mutants in various combinations. Scale bar represents 50 µm (**a**, **d**, **j**) or 100 µm (**g**), respectively. **k**, Quantification of the ratio between the vessel element area and the total xylem area in each mutant. n=6 (wild type), 5 (*max2*), 10 (*max2;smxl6*), 8 (*max2;smxl7*), 8 (*max2;smxl8*), 6 (*max2;smxl6;7*), 12 (*max2:smxl6;8*), 13 (*max2;smxl7;8*), and 7 (*max2;smxl6;7;8*) plants. Statistical groups are indicated by letters and were determined by a one-way ANOVA with post-hoc Tukey-HSD (95 % CI). Source data are provided as a Source Data file.

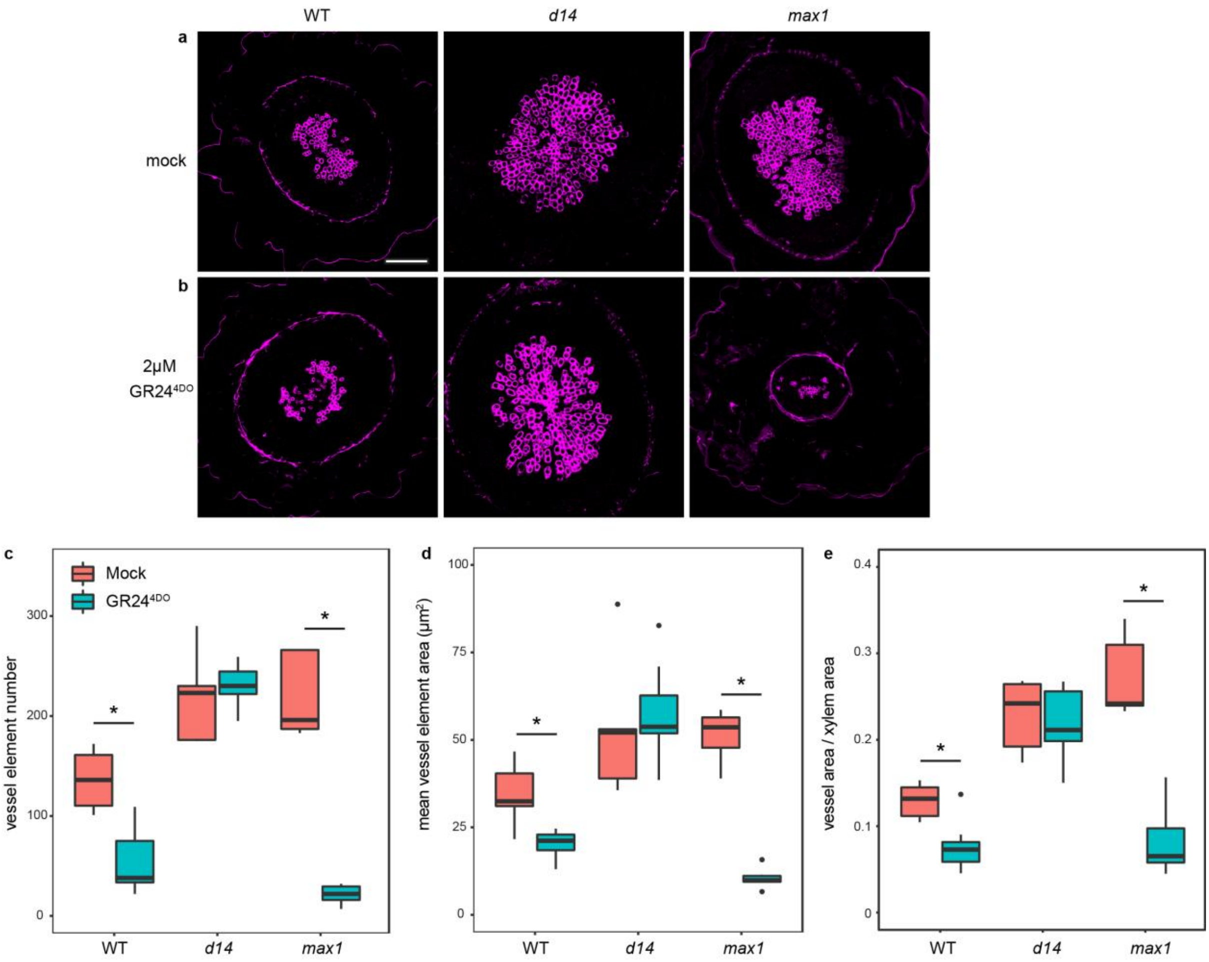

Supplementary 13: Histological analysis of the GR24^{4DO} effect on vessel formation in WT, *d14*, and *max1* mutants.

a, b, Appearance of four week-old plants after application of 2 µM acetone (a) or GR24^{4DO} (b) in wild type, *d14*, and *max1* mutants. Scale bar represents 100 µm. The cross sections were stained with 0.02 % Basic Fuchsin supplemented in ClearSee to stain lignified vessel elements and observed by using 561 nm laser light. Lignified vessel elements were captured and displayed in magenta. The peripheral periderm was stained as well due to suberin deposition. **c**–**e**, Quantification of vessel elements per section (c), mean of individual vessel element area in distinct sections (d) and vessel area / xylem area ratio (e) comparing plants treated with 2 µM acetone and GR24^{4DO}. n=6 (Mock-treated wild type), 8 (GR24^{4DO}-treated wild type), 5 (Mock-treated *d14*), 7 (GR24^{4DO}-treated *d14*), 5 (Mock-treated *max1*), 8 (GR24^{4DO}-treated *max1*) plants. Asterisks indicate $p<0.05$ determined by the two-sided Welch's t-test. *p* values and source data are provided as a Source Data file.

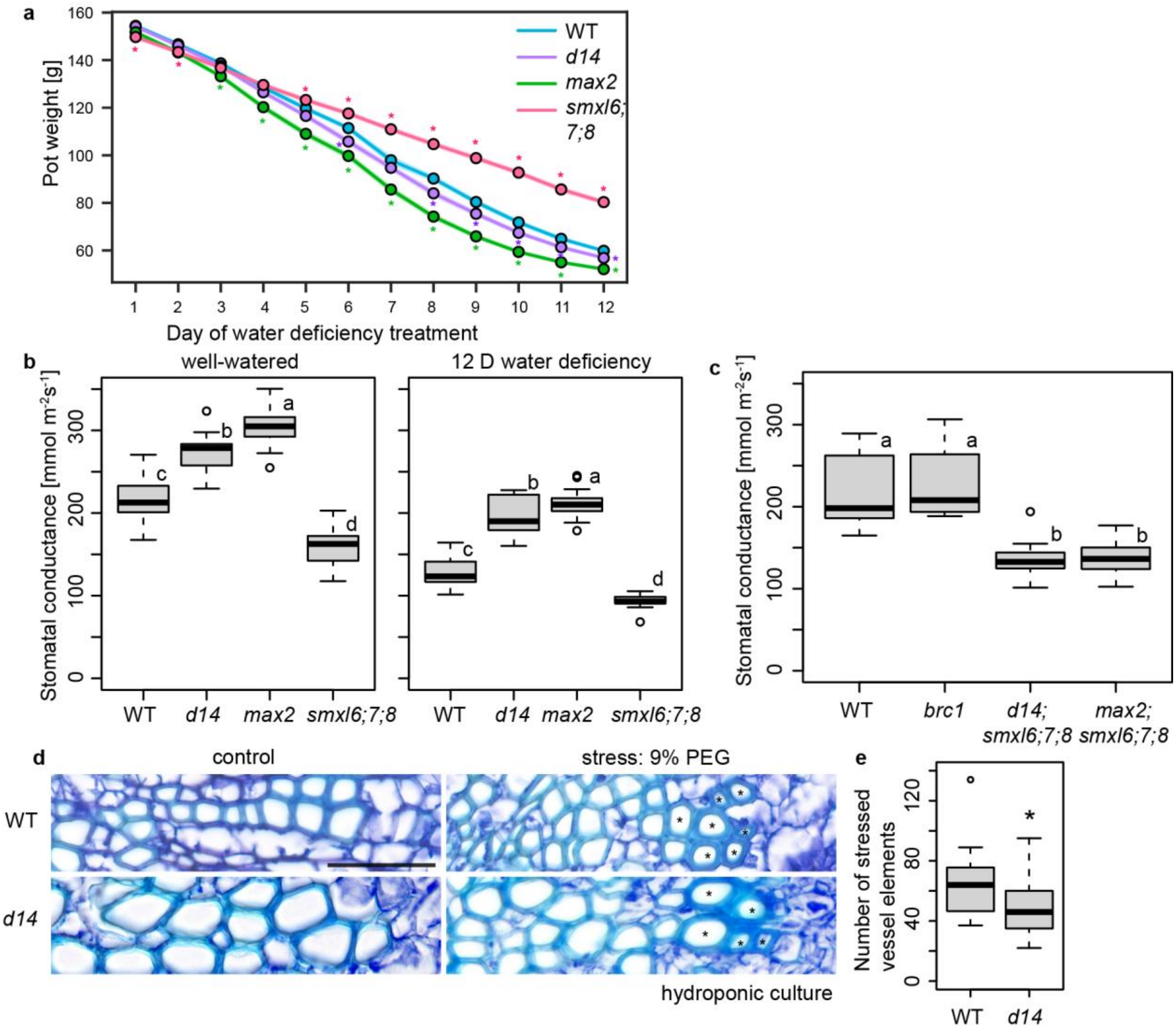

Supplementary Fig. 14: Effect of altered SL-signalling on water usage and stomatal conductance and drought response of vessel elements
a, Pot weight changes when growing wild type (WT), *d14*, *max2* and *smxl6;7;8* plants during water deficiency treatments. Measurements were analysed by t-test at each timepoint comparing mutant genotypes to wild type. Asterisks indicate significance ($p < 0.01$, source Dataset). n=109 (WT), n=111 (*d14*), n=109 (*max2*), n=108 (*smxl6;7;8*). Results from three independent experiments are included. **b**, Quantification and comparison of stomatal conductance found in wild type, *d14*, *max2* and *smxl6;7;8* plants under well-watered conditions. n=18 (wild type, *d14* and *smxl6;7;8*), n=19 (*max2*) and 12 days (12 D) after initiating water deficiency treatments n=18 (wild type and *d14*), n=19 (*max2*), n=17 (*smxl6;7;8*). Conductance was measured on three leaves per plant. Plot shows average conductance of the three leaves per plant. **c**, Stomatal conductance measurement in WT, *brc1*, *d14;smxl6;7;8*, and *max2;smxl6;7;8*. Conductance was measured on three leaves per plant. Plot shows average conductance of the three leaves per plant. n=15 plants each. A *p* value of two-sided Welch's t-test is shown. **d**, Xylem vessel of four-week old WT and *d14* grown under well watered control conditions (left) and subjected to a two-week water deficiency treatment (right) is shown. Asterisks indicate stress related vessels, appearing upon water deficiency. Scale bar indicates 40 µm. **e**, Quantification of the number of stress-related vessel elements in WT and *d14*. n=19 plants (WT), n=22 plants (*d14*). p=0.019 (two-sided Welch's t-test). Statistical groups are indicated by letters and were determined by a one-way ANOVA with post-hoc Tukey-HSD (95% CI). Source data are provided as a Source Data file.

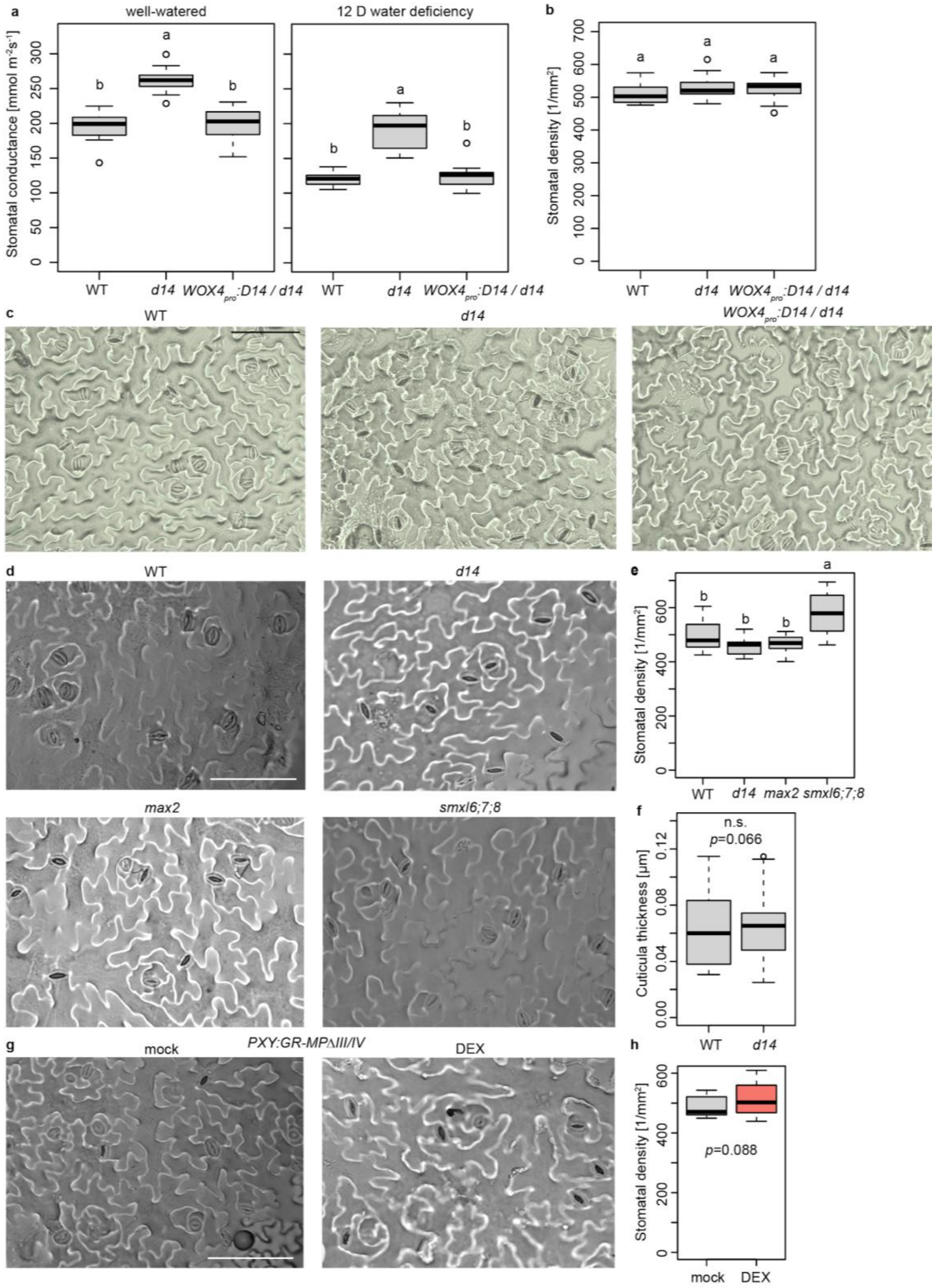

Supplementary Fig. 15: Stomata density analysis in SL-signalling mutants and upon auxin signalling modulation in vascular tissues.
a, Quantification and comparison of stomatal conductance found in wild type, *d14*, and *WOX4*$_{pro}$*:D14/d14* plants under well-watered conditions, n=16 (wild type), n=15 (*d14* and *WOX4*$_{pro}$*:D14/d14*), and 12 days (12 D) after initiating water deficiency treatments, n=17 (wild type), n=15 (*d14* and *WOX4*$_{pro}$*:D14/d14*). Conductance was measured on three leaves per plant. Plots show average conductance of the three leaves per plant. **b**, Stomatal density (stomata per mm^2) comparing genotypes shown in (**c**). n=17 (wild type) n=15 (*d14* and *WOX4*$_{pro}$*:D14/d14*

obtained from three independent experiments. **c**, Photomicrographs of the abaxial leaf surface of the same plants used for the 12 D water deficiency stomatal conductance measurement of wild type, *d14*, and *WOX4*$_{pro}$*:D14/d14* plants. Size bar indicates 50 µm. **d**, Photomicrographs of the abaxial leaf surface of wild type, *d14*, *max2*, and *smxl6;7;8*. Same plants used for stomatal density analysis as for the 12 D water deficiency stomatal conductance measurement. Size bar indicates 50 µm. **e**, Stomatal density (stomata per mm^2) comparing genotypes shown in (**d**). n=16 (wild type and *smxl6;7;8*), n=18 (*max2*), n=17 (*d14*) obtained from three independent experiments. **f**, Quantification of cuticle thickness of WT and *d14*. An average of three measurements from one image is shown. n=13 images from four plants (wild type), n=24 images from six plants (*d14*). A *p* value of Welch's t-test is shown. **g, h**, Photomicrographs and stomatal density on the abaxial leaf surface of mock- or DEXx-treated plants carrying a *PXY*$_{pro}$*:GR-MPΔIII/IV* transgene. n=14 (mock), n=16 (DEX) plants obtained from three independent experiments. *p* value from two-sided Welch's t-test is shown. Statistical groups are indicated by letters and were determined by a one-way ANOVA with post-hoc Tukey-HSD (95 % CI). Size bar indicates 50 µm. Source data are provided as a Source Data file.

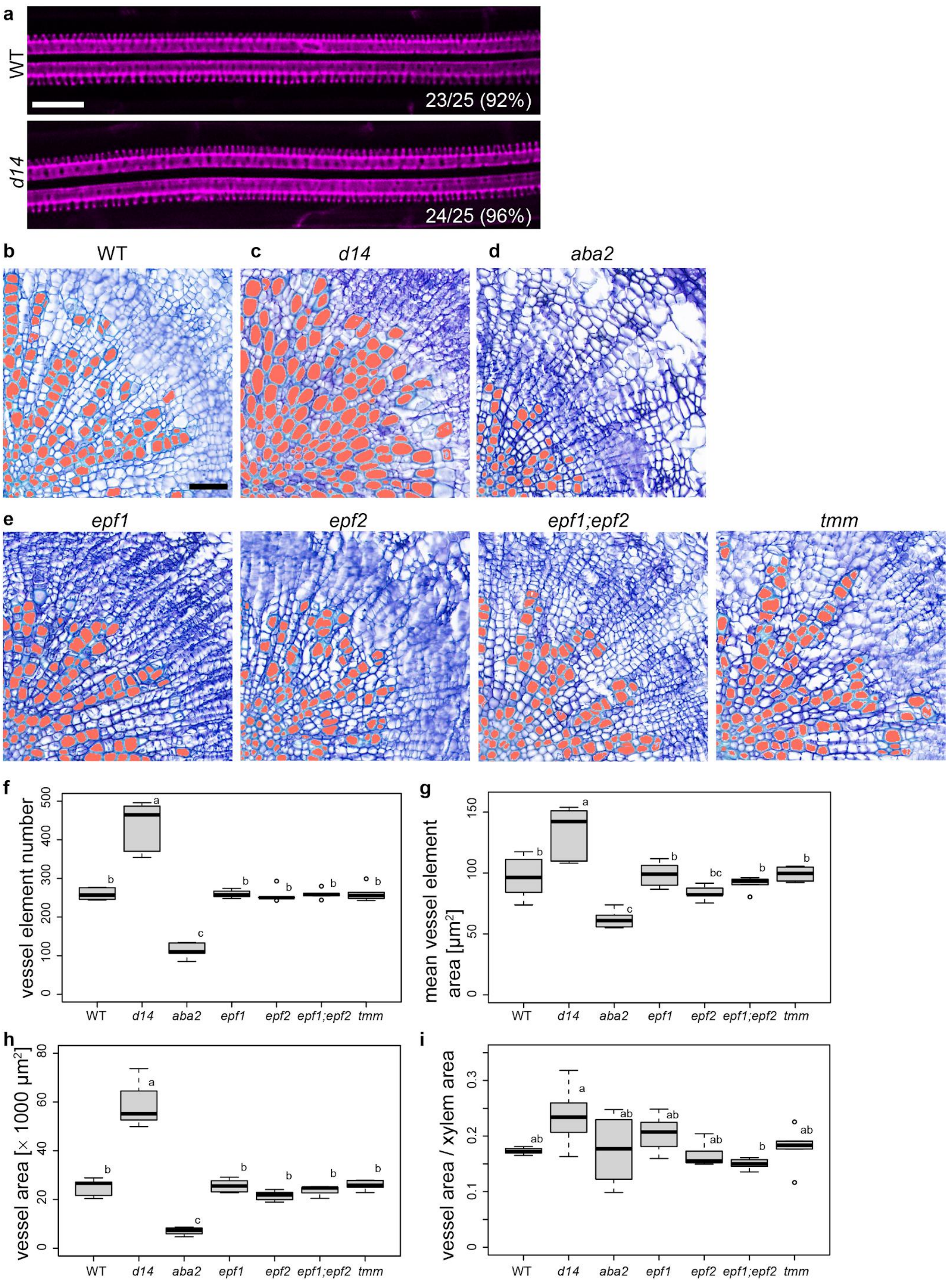

Supplementary Fig. 16: Primary vessel development in *d14* mutants and cambium-derived vessel formation in ABA signalling- and stomata-related mutants.

a, Morphology of xylem strand in wild type and *d14* roots five days after germination. Xylem strands were visualised by Basic Fuchsin staining. The proportion indicates the frequency of plants observed with two metaxylem and two protoxylem strands. The scale bar represents 20 µm. **b**–**e**, Toluidine blue-stained hypocotyl cross-sections from five week-old wild type (WT, **b**) and *d14* (**c**), ABA biosynthesis mutant *aba2-11* (**d**) and stomata-related mutants (**e**)

including *epidermal patterning factor 1* (*epf1*), *epf2*, *epf1;epf2*, and *too many mouths* (*tmm*). Scale bar represents 50 µm. **f**–**i**, Quantification of the vessel element numbers per section (**f**), the average area of individual vessel elements (**g**), the total vessel area per section (**h**) and the ratio between the vessel element area and the total xylem area (**i**) in each mutant shown in (**b**–**e**). n=5 (wild type), 6 (*d14*), 6 (*aba2*), 5 (*epf1*), 5 (*epf2*), 5 (*epf1;epf2*) and 6 (*tmm*). Statistical groups are indicated by letters and were determined by a one-way ANOVA with post-hoc Tukey-HSD (95 % CI). Source data are provided as a Source Data file.
