# Supplementary material for: Strigolactones optimise plant water usage by modulating vessel formation
Source: Nat Commun. 2025 Apr 28;16:3854. doi: 10.1038/s41467-025-59072-y (PMC12037892; doi:10.1038/s41467-025-59072-y)
Supplement: Supplementary file 2 — Description of Additional Supplementary Files [file 41467_2025_59072_MOESM2_ESM.pdf]

Description of Additional Supplementary Files

Supplementary Data 1 Basic statistics of single nucleus (sn) RNA-seq analyses presented in this study.

Supplementary Data 2 Annotation and marker genes detected for each cluster in all the snRNA-seq analyses presented in this study.

Supplementary Data 3 Gene lists used in snRNA-seq analyses obtained from previous studies and the current study.

Supplementary Data 4 Normalised read counts and results of DESeq2 analyses of significantly differentially expressed genes upon DEX induction in $SMXL7_{pro}$:$SMXL7^{d53}$-*GR* (baseMean>50, |log2FoldChange|>0.585, padj<0.01).

Supplementary Data 5 Oligo sequences used in this study.

Supplementary Data 6 Vector construction strategy used in this study.

Supplementary Data 7 Fluorescence signal of each nucleus for each multiwell plate obtained during FANS for VASA-seq analysis.
