# Supplementary material for: Strigolactones optimise plant water usage by modulating vessel formation
Source: Nat Commun. 2025 Apr 28;16:3854. doi: 10.1038/s41467-025-59072-y (PMC12037892; doi:10.1038/s41467-025-59072-y)
Supplement: Supplementary file 10 — Reporting Summary [file 41467_2025_59072_MOESM10_ESM.pdf]

nature portfolio

Corresponding author(s): Dongbo Shi, Thomas Greb

Last updated by author(s): Mar 18, 2025

Reporting Summary

Nature Portfolio wishes to improve the reproducibility of the work that we publish. This form provides structure for consistency and transparency in reporting. For further information on Nature Portfolio policies, see our Editorial Policies and the Editorial Policy Checklist.

Statistics

For all statistical analyses, confirm that the following items are present in the figure legend, table legend, main text, or Methods section.

n/a	Confirmed	
☐	☒	The exact sample size (n) for each experimental group/condition, given as a discrete number and unit of measurement
☐	☒	A statement on whether measurements were taken from distinct samples or whether the same sample was measured repeatedly
☐	☒	The statistical test(s) used AND whether they are one- or two-sided<br>*Only common tests should be described solely by name; describe more complex techniques in the Methods section.*
☒	☐	A description of all covariates tested
☐	☒	A description of any assumptions or corrections, such as tests of normality and adjustment for multiple comparisons
☐	☒	A full description of the statistical parameters including central tendency (e.g. means) or other basic estimates (e.g. regression coefficient) AND variation (e.g. standard deviation) or associated estimates of uncertainty (e.g. confidence intervals)
☐	☒	For null hypothesis testing, the test statistic (e.g. F, t, r) with confidence intervals, effect sizes, degrees of freedom and P value noted<br>*Give P values as exact values whenever suitable.*
☒	☐	For Bayesian analysis, information on the choice of priors and Markov chain Monte Carlo settings
☒	☐	For hierarchical and complex designs, identification of the appropriate level for tests and full reporting of outcomes
☒	☐	Estimates of effect sizes (e.g. Cohen's d, Pearson's r), indicating how they were calculated

Our web collection on statistics for biologists contains articles on many of the points above.

Software and code

Policy information about availability of computer code

Data collection	Leica LAS X, ImageJ (v1.53c), qPCRsoft, BD FACSDiva
Data analysis	Rstudio (v1.4.1106), R (v4.0.4, v4.0.5), STAR (2.7.8a), Cell Ranger (v6.0.1), Seurat (v4.0.6, v4.1.0, v4.3.0), Steel-Dwass test (http://aoki2.si.gunma-u.ac.jp/R/src/Steel-Dwass.R), ggplot2 (v3.3.3), DESeq2 (v1.34.0), rstatix (v0.7.2), g:GOSt (version: e111_eg58_p18_f463989d), Python (v3.10.7), pandas (v1.5.0) and seaborne (v0.12.0), Microsoft Excel 2016<br>Custom codes are deposited at https://github.com/thomasgreb/Zhao-et-al_SL-vessels

For manuscripts utilizing custom algorithms or software that are central to the research but not yet described in published literature, software must be made available to editors and reviewers. We strongly encourage code deposition in a community repository (e.g. GitHub). See the Nature Portfolio guidelines for submitting code & software for further information.

Data

Policy information about availability of data

All manuscripts must include a data availability statement. This statement should provide the following information, where applicable:

- Accession codes, unique identifiers, or web links for publicly available datasets
- A description of any restrictions on data availability
- For clinical datasets or third party data, please ensure that the statement adheres to our policy

Source data for each plot are provided with this paper. The raw sequencing data of snRNA-seq and bulk RNA-seq analyses, and Seurat object files of snRNA-seq data

generated in this study have been deposited at NCBI's Gene Expression Omnibus database under accession code GSE224928 [https://www.ncbi.nlm.nih.gov/geo/query/acc.cgi?acc=GSE224928] or GSE270808 [https://www.ncbi.nlm.nih.gov/geo/query/acc.cgi?acc=GSE270808]. The authors declare that all other data supporting the findings of this study are mentioned in the main text or the supplementary materials.

Research involving human participants, their data, or biological material

Policy information about studies with human participants or human data. See also policy information about sex, gender (identity/presentation), and sexual orientation and race, ethnicity and racism.

Reporting on sex and gender	n/a
Reporting on race, ethnicity, or other socially relevant groupings	n/a
Population characteristics	n/a
Recruitment	n/a
Ethics oversight	n/a

Note that full information on the approval of the study protocol must also be provided in the manuscript.

Field-specific reporting

Please select the one below that is the best fit for your research. If you are not sure, read the appropriate sections before making your selection.

☒ Life sciences ☐ Behavioural & social sciences ☐ Ecological, evolutionary & environmental sciences

For a reference copy of the document with all sections, see nature.com/documents/nr-reporting-summary-flat.pdf

Life sciences study design

All studies must disclose on these points even when the disclosure is negative.

Sample size	No statistical methods were used for sample-size calculation. Sample sizes have been maximized according to practical considerations.
Data exclusions	In snRNA-seq analysis, nuclei with a low number of molecules detected were excluded from further analysis according to the standard pre-established analysis pipeline.
Replication	All the findings were confirmed by usually three but and at least two replicates. snRNA-seq analyses were not been replicated using the exactly same method and type of samples, however, the findings were confirmed in each analysis using different technology and type of samples suggesting their universality.
Randomization	Plant pot positions were randomized in possible cases, however, randomization was not applied to all the experiments due to practical reasons (enhanced physical handling of plants). Covariates were controlled by applying the exact same conditions (growth substrate, temperature, light) to all individuals.
Blinding	Blinding was not possible as phenotypic differences between lines allowed immediate identification.

Reporting for specific materials, systems and methods

We require information from authors about some types of materials, experimental systems and methods used in many studies. Here, indicate whether each material, system or method listed is relevant to your study. If you are not sure if a list item applies to your research, read the appropriate section before selecting a response.

Materials & experimental systems

n/a	Involved in the study	
☒	☐	Antibodies
☒	☐	Eukaryotic cell lines
☒	☐	Palaeontology and archaeology
☒	☐	Animals and other organisms
☒	☐	Clinical data
☒	☐	Dual use research of concern
☐	☒	Plants

Methods

n/a	Involved in the study	
☒	☐	ChIP-seq
☐	☒	Flow cytometry
☒	☐	MRI-based neuroimaging

Dual use research of concern

Policy information about dual use research of concern

Hazards

Could the accidental, deliberate or reckless misuse of agents or technologies generated in the work, or the application of information presented in the manuscript, pose a threat to:

No	Yes	
☒	☐	Public health
☒	☐	National security
☒	☐	Crops and/or livestock
☒	☐	Ecosystems
☒	☐	Any other significant area

Experiments of concern

Does the work involve any of these experiments of concern:

No	Yes	
☒	☐	Demonstrate how to render a vaccine ineffective
☒	☐	Confer resistance to therapeutically useful antibiotics or antiviral agents
☒	☐	Enhance the virulence of a pathogen or render a nonpathogen virulent
☒	☐	Increase transmissibility of a pathogen
☒	☐	Alter the host range of a pathogen
☒	☐	Enable evasion of diagnostic/detection modalities
☒	☐	Enable the weaponization of a biological agent or toxin
☒	☐	Any other potentially harmful combination of experiments and agents

Plants

Seed stocks

All plant lines used in this study were Arabidopsis thaliana (L.) Heynh. plants of the accession Columbia (Col-0). Sources of more specific plant material are described in the Methods section.

Novel plant genotypes

Novel transgenic lines were generated through the floral dipping method using Agrobacterium tumefaciens. More details are provided in the Methods section.

Authentication

Obtained and newly generated transgenic lines were authenticated by PCR on genomic DNA applying informative and distinct primer combinations for amplification. To avoid misinterpretation of genetic effects due to second-site mutations, independent mutants were analyzed in which different components of investigated pathways were disrupted, whenever possible.

Flow Cytometry

Plots

Confirm that:

☒ The axis labels state the marker and fluorochrome used (e.g. CD4-FITC).

☒ The axis scales are clearly visible. Include numbers along axes only for bottom left plot of group (a 'group' is an analysis of identical markers).

☒ All plots are contour plots with outliers or pseudocolor plots.

☒ A numerical value for number of cells or percentage (with statistics) is provided.

Methodology

Sample preparation

Hypocotyls were dissected and collected in petri dishes incubated on ice. 2 ml of 1x nuclei isolation buffer (CelLytic ™ PN Isolation/Extraction Kit, Sigma #CELLYTPN1) supplemented with 20 µl RiboLock RNase inhibitor 40 U/µL (ThermoFisher #EO0381) and Hoechst 33342 at 10 µg/ml final concentration were prepared and a minimum amount of buffer was applied to submerge the collected hypocotyls. Hypocotyls were chopped using razor blades (Wilkinson Sword) for up to 5 min and

	transferred on a gentle shaker at 4°C for 15 min. Samples were then filtered through a 50 µm filter (CellTrics, Sysmex #04-004-2327) and passed to a low protein binding tube (Eppendorf #0030108132). A sheath pressure of 35 psi and a drop drive frequency of 60 kHz were applied.
Instrument	BD FACSAriaTM IIIu cell sorter
Software	BD FACSDiva™
Cell population abundance	Single nuclei were sorted in each well of multi-well plate for VASA-seq analysis, or 50,000 nuclei were sorted in a sample tube for 10x Chromium analysis. Nuclei concentration was determined using fluorescence microscopy.
Gating strategy	Gating strategy is described in Extended Data Figure 1. The signature of endoduplication in Hoechst staining was used to set the gates for the nuclei. The gate for GFP+ and/or RFP+ nuclei was set using wild type as a reference.

☒ Tick this box to confirm that a figure exemplifying the gating strategy is provided in the Supplementary Information.
