# Supplementary material for: Strigolactones optimise plant water usage by modulating vessel formation
Source: Nat Commun. 2025 Apr 28;16:3854. doi: 10.1038/s41467-025-59072-y (PMC12037892; doi:10.1038/s41467-025-59072-y)
Supplement: Supplementary file 11 — Transparent Peer Review file [file 41467_2025_59072_MOESM11_ESM.pdf]

nature portfolio

Peer Review File

Strigolactones optimise plant water usage by modulating vessel formation

Corresponding Author: Professor Thomas Greb

This manuscript has been previously reviewed at another journal. This document only contains information relating to versions considered at Nature Communications.

This file contains all reviewer reports in order by version, followed by all author rebuttals in order by version.

Version 0:

Reviewer comments:

Reviewer #1

(Remarks to the Author)
I am largely satisfied with the improvement of the manuscript and the authors' response to my comments. New data and analyses have greatly improved the quality of the research. This study reports novel roles for strigolactone signaling in vessel element formation and water use efficiency in response to drought, and reveals a new strategy for drought resistance improvement by manipulating vascular development. Although the experiments on drought regulation of SL content and SL biosynthesis gene expression did not yield the desired results, I support the publication of this study in Nature Communications, considering its innovation and importance.

(Remarks on code availability)

Open Access This Peer Review File is licensed under a Creative Commons Attribution 4.0 International License, which

permits use, sharing, adaptation, distribution and reproduction in any medium or format, as long as you give appropriate credit to the original author(s) and the source, provide a link to the Creative Commons license, and indicate if changes were made.
In cases where reviewers are anonymous, credit should be given to 'Anonymous Referee' and the source.
The images or other third party material in this Peer Review File are included in the article's Creative Commons license, unless indicated otherwise in a credit line to the material. If material is not included in the article's Creative Commons license and your intended use is not permitted by statutory regulation or exceeds the permitted use, you will need to obtain permission directly from the copyright holder.
To view a copy of this license, visit https://creativecommons.org/licenses/by/4.0/
